# HIV transgenic mouse monocytes display increased *in vivo* migration across the blood-brain barrier associated with increased expression of genes associated with mononuclear leukocyte movement

**DOI:** 10.1128/jvi.02063-25

**Published:** 2026-04-20

**Authors:** Agnes Sydenstricker, Danica Lee, Adilyn Voss, Jian Hua Zheng, Hong Hur, Manoj Kandpal, Harris Goldstein

**Affiliations:** 1 Department of Microbiology & Immunology, Albert Einstein College of Medicine 2006, Bronx, New York, USA; 2 Center for Clinical and Translational Science, Rockefeller University Hospital, New York, New York, USA; 3 Department of Pediatrics, Albert Einstein College of Medicine, Bronx, New York, USA; University Hospital Tübingen, Tübingen, Germany

**Keywords:** human immunodeficiency virus, blood-brain barrier, monocytes

## Abstract

Soon after HIV acquisition, circulating HIV-infected monocytes cross the blood-brain barrier (BBB) and infect resident brain microglia and other susceptible cells, establishing a potential viral reservoir. Despite suppressive antiretroviral therapy, these HIV-infected cells mediate a neuroinflammatory process causing HIV-associated neurocognitive deficits (HAND) in at least 20% of people with HIV. Continued migration of HIV-infected monocytes into the brain may further exacerbate neuroinflammation and replenish viral reservoirs. To ascertain how HIV infection facilitates monocyte passage across the BBB *in vivo*, we developed a novel mouse model to quantify circulating monocytes supporting HIV production that migrated into the brain. We demonstrate that significantly more monocytes from HIV-transgenic mice, capable of supporting HIV production, crossed the BBB compared to control transgenic mouse monocytes. This difference was particularly pronounced after recipient mice were treated with lipopolysaccharide (LPS). To explore the underlying mechanism, we compared the transcriptomes of HIV transgenic mouse monocytes and control mouse monocytes and identified multiple differentially expressed genes linked to mononuclear leukocyte trafficking, including several associated with monocyte chemotaxis. We also evaluated the effect of substance use in combination with HIV infection on monocyte migration across the BBB into the brain by treating HIV transgenic mice with either morphine or methamphetamine. Short-term exposure to either drug did not significantly alter the migration of HIV-transgenic monocytes across the BBB.

**IMPORTANCE:**

Over 20% of people with HIV (PWH) develop cognitive and neurological deficits despite antiretroviral therapy. While the blood-brain barrier (BBB) normally prevents brain entry of circulating monocytes, HIV enables infected monocytes to traverse the BBB, establish viral production within the brain, and subsequently infect microglia and other cells, which drives neuroinflammation, neuronal injury, and neurocognitive impairment. To investigate how HIV stimulates *in vivo* migration of circulating monocytes across the BBB, we developed a novel mouse model utilizing HIV-transgenic mouse monocytes that support HIV production. After intravenous injection, a significantly higher number of HIV-transgenic monocytes migrated into the brains of wild-type mice compared to control transgenic monocytes, particularly after lipopolysaccharide (LPS) treatment. We identified multiple genes differentially expressed in HIV-transgenic monocytes associated with mononuclear leukocyte trafficking linked to HIV-mediated induction of monocyte transmigration across the BBB. These genes may represent therapeutic targets to prevent HIV-infected monocyte migration into the brain.

## INTRODUCTION

Following HIV acquisition, circulating HIV-infected monocytes migrate across the blood-brain barrier (BBB) into the brain (1, 2). Within the brain, HIV produced by these cells infects resident microglia and other susceptible cells, initiating neuroinflammatory processes and potentially leading to the subsequent development of HIV-associated neurocognitive deficits (HAND) (1–5). The incidence of HAND is increased among people living with HIV (PWH) who exhibit elevated levels of circulating mature CD14+ CD16+ monocytes (1, 3, 6–8) and a greater HIV burden within these monocytes (9–13). Once within the brain, HIV-infected monocytes may transmit infection to microglia and differentiate into latently infected macrophages capable of intermittent HIV production, thereby contributing to subsequent neurodegeneration and CNS damage (2). Despite treatment with antiretroviral therapy (ART), circulating HIV-infected monocytes may persist and continue to migrate across the BBB into the brain and amplify the chronic, low-level inflammation and neuronal damage that leads to cognitive impairment (11). Furthermore, substance use disorder (SUD), a common comorbidity in PWH, has been reported to increase neuroinflammation and may increase the incidence of HAND (14–16). Understanding how HIV infection alone or in combination with substances of abuse affects monocyte migration across the BBB and how this process contributes to the chronic replenishment of viral reservoirs in the brain has the potential to inform therapeutic developments aimed at preventing the influx of HIV-infected monocytes and, ultimately, the onset of HAND. However, the inability to monitor the *in vivo* migration of HIV-infected monocytes across the BBB in PWH limits our understanding of the mechanisms by which HIV infection alone, and in combination with abused substances, increases the trafficking of circulating monocytes across the BBB.

To investigate the mechanisms by which HIV infection alone and in combination with other factors, such as lipopolysaccharide (LPS), opioids, and methamphetamine, affect the *in vivo* capacity of monocytes to cross the BBB, we developed a novel mouse model that permits quantification of the number of circulating HIV-infected monocytes that migrate into the brain. Our transgenic mouse model circumvented the inability of HIV to infect mouse cells due to species-specific replication blocks. These include the inability of the murine homologs of CD4 and CCR5, the primary HIV receptor and coreceptor, to bind HIV envelope and enable viral entry (17) and of the murine homolog of cyclin T1 to bind HIV Tat and recruit the positive transcription elongation factor b (P-TEFb) complex to the HIV-1 TAR RNA target element to generate the HIV transcripts essential for efficient HIV replication (18–20). This transgenic mouse model bypassed the viral entry block by expressing a transgene containing a full-length HIV provirus regulated by its endogenous long terminal repeat (LTR) and the Tat function block by expressing a transgene consisting of human cyclin T1 regulated by a CD4 promoter/enhancer (JRhCyct mice). The CD4-specific expression of human cyclin T1 circumvented the HIV Tat block in CD4-expressing cells and enabled *in vivo* HIV production by CD4 T cells and monocytes in mice also transgenic for an HIV transgene, validated by the development of high levels of viremia in these JRhCyct mice (21, 22). We also generated mice expressing the human CD4, CCR5, and cyclin T1 transgenes under the control of a CD4 enhancer/promoter (CCC mice), enabling mouse CD4 T cells and monocytes to be infectible with HIV as demonstrated by the development of disseminated infection after intravenous (IV) or mucosal inoculation of CCC mice with HIV (23). We crossed these two transgenic mouse models to generate mice (JRCCC mice) that are transgenic for a full-length HIV provirus regulated by its endogenous LTR and for human CD4, CCR5, and cyclin T1, regulated by a CD4-promoter/enhancer. JRCCC mice developed HIV viremia at levels (>10^5^ copies of HIV RNA/mL) comparable to levels observed in untreated PWH. In the current study, we demonstrated the successful application of our JRCCC HIV transgenic mice for the establishment of a model to investigate the *in vivo* effect of HIV infection alone and in combination with substances of abuse on the migration of circulating HIV-infected monocytes across the BBB. In addition, we used this model to identify genes upregulated in the HIV transgenic mouse monocytes that may play a role in enhancing their passage across the BBB.

## RESULTS

### Design and construction of transgenic mice expressing a full-length HIV provirus and human CD4, CCR5, and cyclin T1

**Fig 1:**
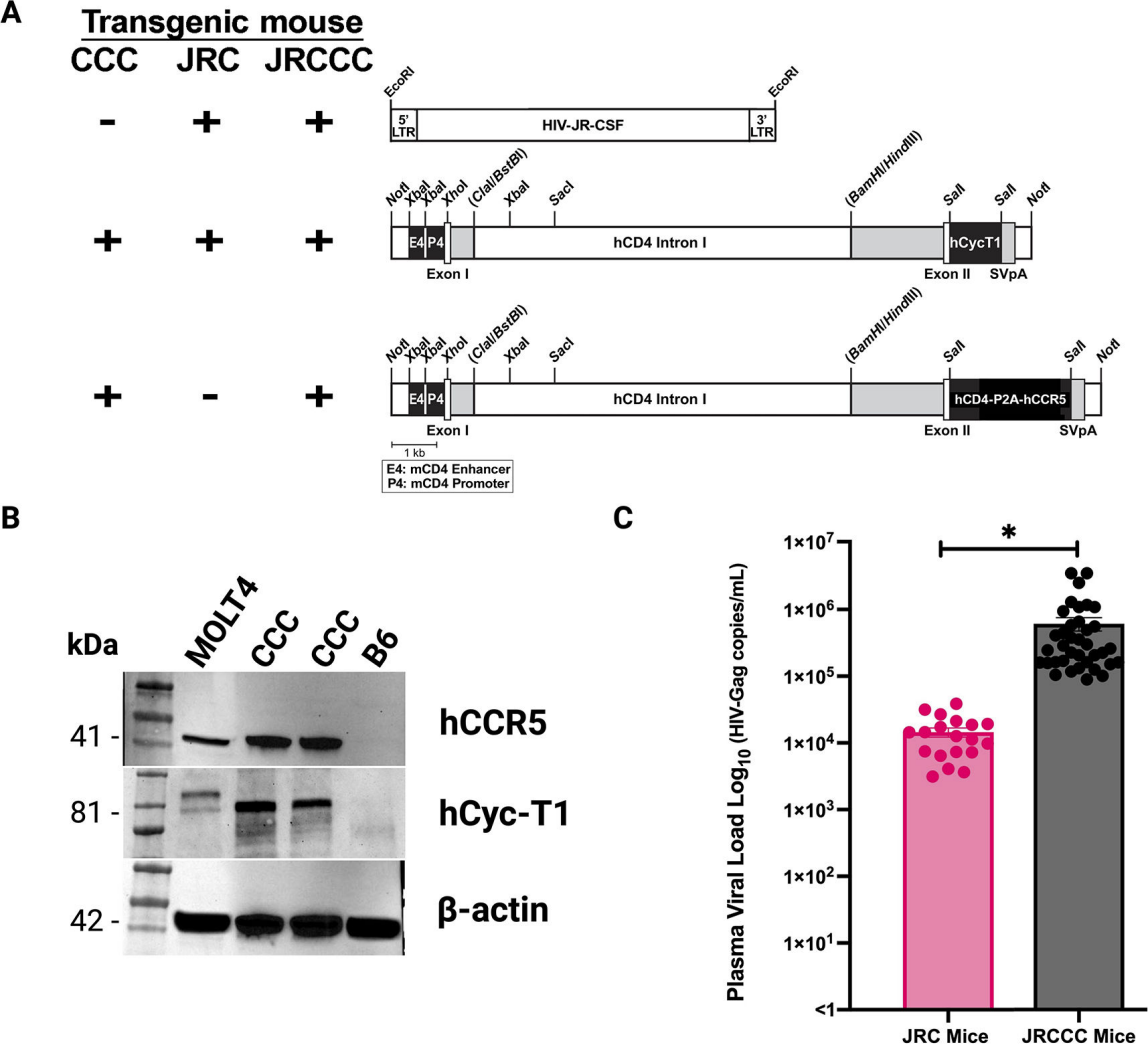
Construction of JR-CSF/hCD4/CCR5/cT1 mice and evaluation of transgene expression. (**A**) Panel represents the transgene constructs present in each of the three transgenic mouse lines (left). Schematic representation of the HIV-JR-CSF transgene construct, including the LTR regulatory domain with restriction enzyme sites for EcoRI/StuI and the human cyclin T1, and human CD4/CCR5 transgene construct, including the murine E4/P4 CD4 enhancer/promoter and restriction enzyme sites for NotI (right). (**B**) Western blots demonstrated the detection of human CCR5 and Cyclin-T1 in CCC transgenic mice splenocytes but not in B6 mice. MOLT4-CCR5 cells provide a positive control for human CCR5 expression. (**C**) Plasma viral loads of JRC mice (*n* = 19 mice) compared to JRCCC mice (*n* = 37 mice) were determined using a quantitative HIV-Gag-specific RT-qPCR assay. The bar graph displays the viral load for individual mice with the mean ± SEM of HIV-Gag copies/mL displayed (*P ≤ 0.05).

We constructed three vectors to generate a mouse line transgenic for the tightly linked transmission of an HIV provirus under the control of its endogenous HIV LTR and human CD4, CCR5, and cyclin T1 genes, regulated by a murine CD4 expression cassette that targets expression to CD4 T lymphocytes, macrophages, and dendritic cells (Fig. 1A) (22, 23). The first vector is a full-length HIV-1 provirus molecular clone derived from the well-characterized primary R5-tropic clinical isolate HIV-1_JR-CSF_ (24). The second vector consists of the human cyclin T1 gene regulated by a murine CD4 expression cassette as previously described (22). The third vector was constructed by linking the human CD4 and human CCR5 gene with a ‘‘self-cleaving’’ P2A peptide expressed as a single transcript regulated by a murine CD4 expression cassette to produce equimolar concentrations of human CD4 and CCR5 as described (23). Co-injecting these three constructs into fertilized mouse oocytes produced founder mice with either tandem integration of the HIV-1_JR-CSF_ provirus and human cyclin T1 constructs (JRC mice) or all three constructs (JRCCC mice) with transmission of these transgenes as a single allele. Human CCR5 and cyclin-T1 were detected in the transgenic mouse splenocytes by western blot, indicating their expression (Fig. 1B). *In vivo* HIV production was demonstrated by the development of HIV viremia in transgenic mice. While none of the JRC mice had plasma levels >10^5^ HIV RNA copies/mL, the majority of the JRCCC mice did have plasma levels >10^5^ HIV RNA copies/mL. The mean level of HIV RNA in the plasma of JRCCC mice (611,014 ± 138,440 HIV RNA copies/mL) was significantly higher (*P ≤* 0.01) than the viral loads of JRC mice (mean = 14,486 ± 2,247), which are transgenic for only the JRCSF provirus and the CD4 promoter-regulated human cyclin T1 gene (Fig. 1C) (22). While we observed increased *in vivo* HIV viremia in the JRCCC mice compared to the JRC mice, we cannot definitively attribute this effect to productive infection of mouse monocytes within the animals.

### Development of a mouse model to quantify the *in vivo* migration of monocytes across the BBB

Multiple studies have indicated that circulating HIV-infected monocytes are the primary cells that migrate across the BBB and introduce HIV infection into the brain (1, 2, 4). We postulated that we could use monocytes supporting HIV production from our JRCCC transgenic mice to develop an *in vivo* model to investigate the mechanisms by which HIV infection alone and in combination with other factors enhances the *in vivo* capacity of circulating HIV-producing monocytes to cross the BBB into the brain. For this purpose, we intravenously injected JRCCC monocytes into syngeneic C57BL/6 (B6) mice and quantified their passage across the BBB into the brain. Monocyte passage across the BBB in PWH may be facilitated by elevated levels of LPS present in their plasma due to bacterial translocation across a compromised gut barrier; the increased LPS in the blood may induce systemic inflammation, increase the BBB permeability, and activate HIV transcription in HIV-infected monocytes (25–27). Previous studies have shown a significant increase in HIV-gp120 migration in mice occurring at 6 h after IP LPS injection (28) and microglial hyperactivity 4 h after IP LPS injection (29). Using this study as a starting point, we determined that 4 h after LPS treatment recapitulated the inflammatory milieu required to facilitate migration of intravenously injected mouse monocytes into the mouse brain and used this time period for our subsequent LPS treatment experiments.

**Fig 2:**
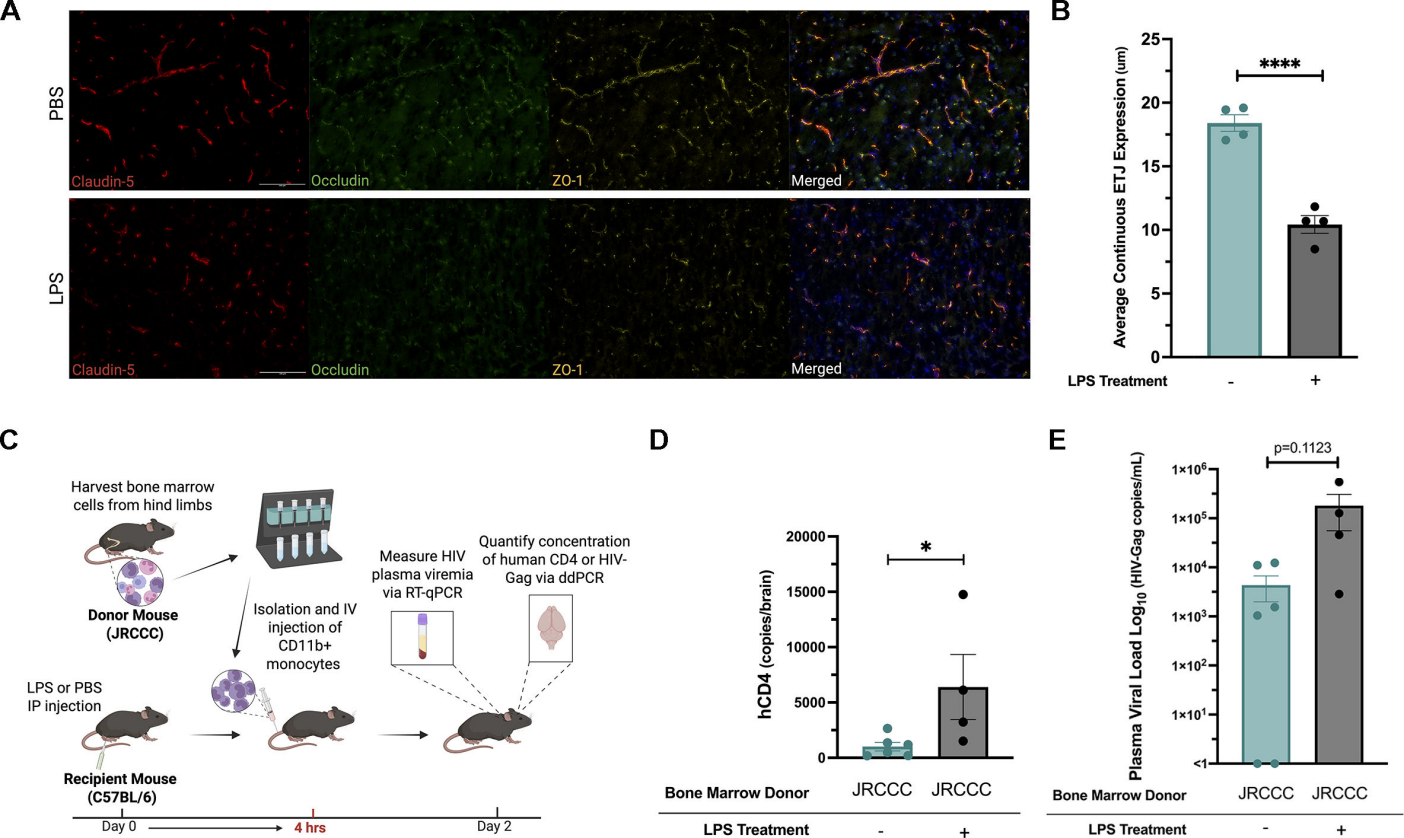
Increased transmigration of HIV transgenic monocytes into the brains of recipient mice. (**A**) Evaluation of BBB integrity by immunofluorescent visualization of the ETJ proteins claudin-5, occludin, and ZO-1 in cerebral arteries 4 h after B6 mice were injected intraperitoneally with either PBS or LPS (3 mg/kg). Coronal brain sections (15-µm thick) were stained for cell nuclei (blue), claudin-5 (red), occludin (green), and ZO-1 (yellow), and were imaged at 20× magnification (scale bar = 100 µm). (**B**) Continuity of ETJ protein expression was determined by measuring the average length of blood vessels continuously stained for ETJ proteins in four different fields in similar hypothalamic regions of the mouse brain tissue for PBS and LPS-treated mice. Each symbol in the bar graph represents an average continuous ETJ expression in one visual field, with mean ± SEM shown, and the data are representative of two experiments, each done with *n* = 2 mice. Statistical analysis was performed using unpaired *t*-tests between groups. (**C**) Experimental set-up and timeline. Monocytes, harvested from JRCCC bone marrow leukocytes (BML) and highly purified by immunomagnetic sorting, were injected intravenously (20 × 10^6^ monocytes/mouse) into the tail veins of B6 mice 4 h after they were intraperitoneally injected with LPS (3 mg/kg, *n* = 4 mice) or PBS (*n* = 6 mice). (**D**) DNA from harvested mouse brains was evaluated for the number of human CD4 copies by droplet digital PCR (ddPCR), which represents the presence of individual JRCCC monocytes. Each symbol represents the number of human CD4 copies detected by ddPCR in the brain of an individual mouse. The individual and average number of human CD4 DNA in copies/brain for PBS or LPS-treated mice are shown with mean ± SEM (*P ≤ 0.05). (**E**) Two days after the highly purified JRCCC monocytes were injected intravenously into B6 mice, the mice were bled to quantify the level of HIV viremia by RT-qPCR. The data represent pooled data from five experiments, with each symbol representing the number of HIV RNA copies/mL in individual mice. The individual and average viral loads with mean ± SEM of HIV-Gag RNA copies/mL in the plasma of PBS or LPS-treated mice are shown.

We confirmed BBB compromise 4 h after LPS treatment by evaluating blood vessel integrity of untreated and LPS-treated mice using immunofluorescent staining to visualize continuity of the endothelial tight junction (ETJ) proteins claudin-5, occludin, and ZO-1, which are required for BBB function (30). Four hours after B6 mice were intraperitoneally injected with either LPS (3 mg/kg) or PBS, the mice were intracardially perfused sequentially with PBS and 4% paraformaldehyde (PFA). The brains were harvested, embedded in OTC, sectioned, and stained with antibodies to the ETJ proteins claudin-5, occludin, and ZO-1 for immunofluorescent visualization (Fig. 2A). Based on next-generation HIV proviral sequencing, HIV-infected cells are present throughout the brain (31); thalamic and hypothalamic regions are well-vascularized; therefore, these regions were selected for ETJ quantification. Examination of the stained sections indicated multiple gaps in ETJ protein expression throughout the brain regions of LPS-treated mice compared to brain sections in untreated mice. The continuity of claudin-5, occludin, and ZO-1 in the vascular ETJs was analyzed and quantified using the ImageJ FIJI program (32) and AnalyzeSkeleton plugin (33) and demonstrated that LPS treatment reduced the continuous expression of claudin-5, occludin, and ZO-1 in blood vessels by 43% in the brain sections from PBS-treated mice (mean = 18.4 ± 1.3 um) compared to the brain sections from LPS-treated mice (mean = 10.4 µM ± 1.4 um) (Fig. 2B).

We investigated if we could quantify the migration of HIV-expressing monocytes across the LPS-compromised BBB using a droplet digital PCR (ddPCR) assay specific for the human CD4 transgene as outlined in the experimental protocol (Fig. 2C). Specific DNA targets, such as the human CD4 transgene, can be quantified down to single copy levels by ddPCR by using sample partitioning during PCR (34) as described to quantify the number of HIV-infected cells in the brains of PWH (35, 36). We designed a custom ddPCR assay using primer pairs and a probe specific for the human CD4 transgene to selectively quantify the number of infused JRCCC or CCC monocyte cells that migrated across the BBB into the brain. After highly purified monocytes (~98% CD11b+) from JRCCC mouse bone marrow leukocytes (BML) were obtained by immunomagnetic sorting using CD11b MicroBeads (Miltenyi Biotec), they were intravenously injected (20 × 10^6^ cells/mouse) into B6 mice 4 h post-LPS or PBS treatment. Two days later, the mice were perfused with PBS, after which their whole brains were harvested for DNA extraction. The number of JRCCC monocytes that had migrated across the BBB into the brain was then quantified by ddPCR. While JRCCC monocytes were detected by human CD4-specific ddPCR in the brains of both PBS and LPS-treated B6 mice, the brains of the LPS-treated mice contained >6-fold higher numbers of JRCCC monocytes (mean = 6,403 ± 2,941 copies/brain) than the brains of PBS-treated mice (mean = 1,014 ± 385 copies/brain) (Fig. 2D). Two days after intravenous injection with the JRCCC monocytes, plasma viremia was detected in all of the LPS-treated B6 mice (mean = 181,212 ± 125,382 copies/mL) and four of the six PBS-treated B6 mice (mean = 4,333 ± 2,350 copies/mL) (Fig. 2E), indicating that active HIV production was occurring in the JRCCC monocytes after transfer into the B6 mice and was increased by over 40-fold by LPS treatment of the recipient B6 mice. Collectively, these results indicate that JRCCC monocytes not only support active *in vivo* HIV production but can also cross the intact BBB. The number of migrating monocytes was significantly enhanced in LPS-treated B6 mice, likely B6 mice, likely reflecting both LPS-induced compromise of BBB integrity in the recipient mice and stimulation of HIV production by the transferred JRCCC monocytes.

### Expression of HIV enhances *in vivo* migration of JRCCC bone marrow leukocytes across the BBB into the brain

**Fig 3:**
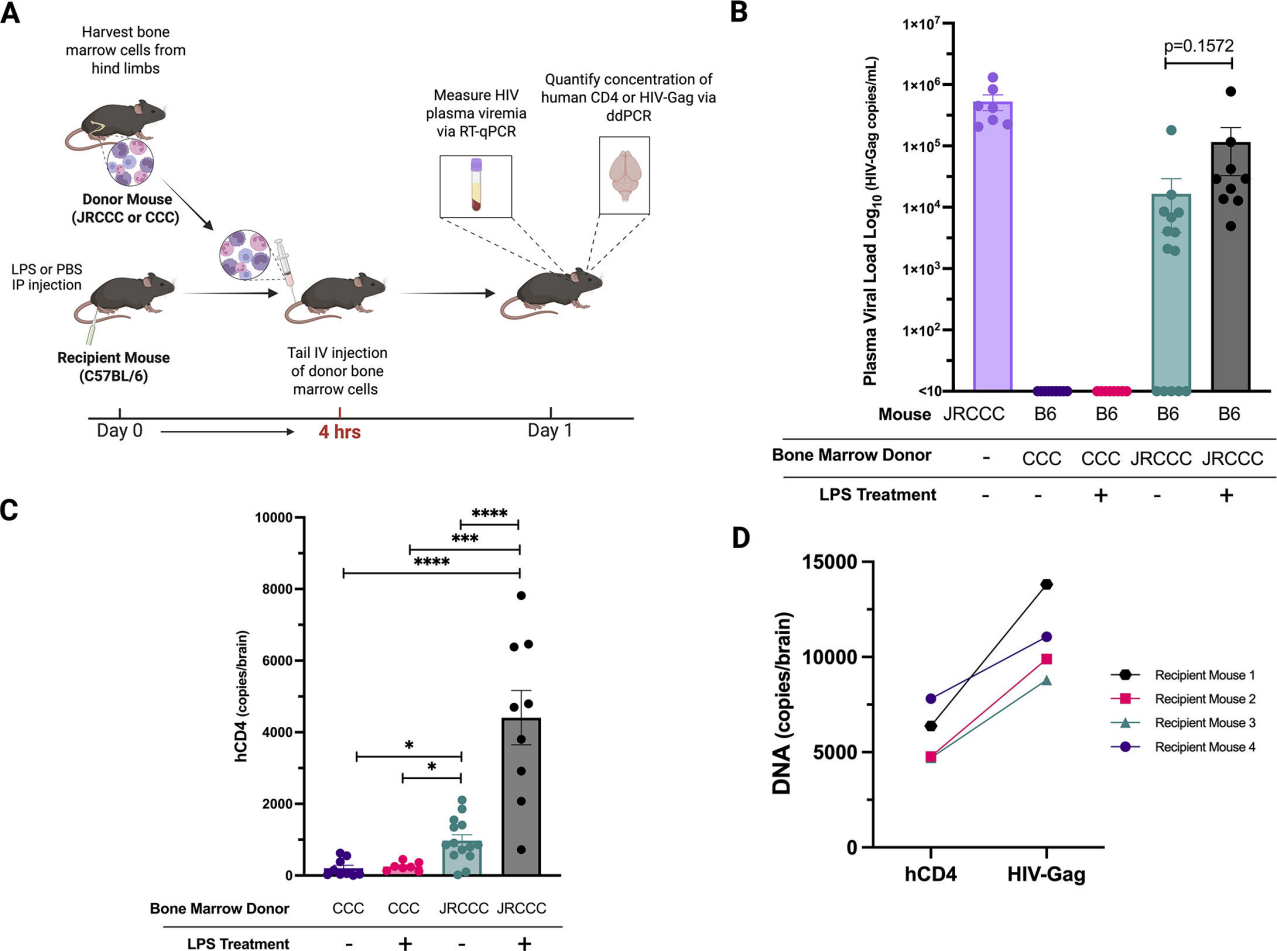
Increased transmigration of JRCCC BML across the BBB compared to CCC BML. (**A**) Experimental set-up and timeline. BML was harvested from JRCCC or CCC mice and intravenously injected into B6 mice 4 h after they were treated with 3 mg/kg LPS (*n* = 15 mice) or PBS (*n* = 16 mice). (**B**) Pooled HIV plasma viremia data from six experiments, with each data point representing HIV-Gag copies/mL for individual mice, are shown in a bar graph with mean ± SEM. (**C**) Pooled ddPCR data from six experiments with brain DNA samples normalized to 25 ng/µL DNA are shown in a bar graph with mean ± SEM, and each symbol represents the number of human CD4 DNA copies/brain of individual mice. Statistical analysis was performed using unpaired *t*-tests between groups (****P ≤ 0.0001, ***P ≤ 0.001, *P ≤ 0.05). (**D**) The indicated brain DNA samples were analyzed using ddPCR using primer pairs and probes for quantifying HIV-Gag copies/brain and human CD4 copies/brain, with each point representing data from an individual mouse, and the HIV-Gag copies/brain and human CD4 copies/brain from the same mouse are indicated by unique markers and the connecting line.

After validating the model with highly purified monocytes and confirming increased migration of JRCCC monocytes across the BBB, subsequent studies were conducted with unfractionated JRCCC or CCC BML. The majority of BMLs are monocytes (>60% CD11b+) and using unfractionated BMLs avoids cell loss and potential artifacts incurred during immunomagnetic sorting. To examine the role of HIV production on the capacity of JRCCC BML migration across the BBB, we compared the number of circulating JRCCC BML to CCC BML that migrated into the brains of untreated and LPS-treated mice as outlined in the experimental protocol (Fig. 3A). B6 mice were intravenously injected with either JRCCC BML (20 × 10^6^ cells) or CCC BML (20 × 10^6^ cells) 4 h after intraperitoneal injection of either PBS or LPS. Two days later, plasma viremia was detected in all of the LPS-treated B6 mice (9 of 9, mean = 115,653 HIV copies/mL) and in 64% of the PBS-treated B6 mice (9 of 14, mean = 16,533 HIV copies/mL) as compared to the plasma viremia present in the donor JRCCC mice (mean = 527,970 HIV copies/mL) (Fig. 3B). This indicated active viral production by the transferred JRCCC BML. The mice were sacrificed, intracardially perfused with PBS, and their whole brains were harvested. Following DNA extraction, human CD4 copy number was quantified using the human CD4 ddPCR assay (Fig. 3C). In the absence of LPS treatment, donor JRCCC BML were detected in 85% (12 of 14) of the B6 mouse brains compared to 33% (3 of 9) of the brains of B6 mice injected with CCC BML; a 4.7-fold higher (*P* = 0.0019) mean number of JRCCC BML was detected in the brains of B6 mice (mean = 973 ± 612 copies/brain) compared to mice injected with CCC BML (mean = 205 ± 247 copies/brain). LPS treatment of B6 mice did not result in a significant increase in the number of CCC BML migrating across the BBB into the brain (mean = 251 ± 119 copies/brain) compared to the brains of PBS-treated mice (mean = 205 ± 247 copies/brain). In contrast, LPS treatment of B6 mice significantly increased (*P ≤* 0.0001) the number of circulating JRCCC BML which migrated into the brain by ~4.5-fold (mean = 4,407 ± 2,280 copies/brain) as compared to the brains of PBS-treated B6 mice (mean = 973 ± 612 copies/brain). Furthermore, the number of JRCCC BML detected in the brains of LPS-treated B6 mice (mean = 4,407 ± 2,280 copies/brain) was ~17.5-fold higher (*P* = 0.0003) compared to the brains of LPS-treated B6 mice injected with CCC BML (mean = 251 ± 119 copies/brain). To validate the quantification of the JRCCC BML that migrated into the brain using the human CD4-specific ddPCR, we selected representative brains from LPS-treated mice injected with JRCCC BML for quantification by ddPCR using primer pairs and a probe specific for the HIV-Gag sequence. We observed comparable levels of JRCCC BML detected in the brains from LPS-treated B6 mice (*n* = 4 mice) by the HIV Gag-specific ddPCR assay compared to the human CD4-specific ddPCR with some increased sensitivity for the HIV-Gag primer pair and probes (Fig. 3D).

### Differential expression of genes associated with mononuclear leukocyte movement by monocytes from PBS- or LPS-treated JRCCC mice compared to CCC mice

**Fig 4:**
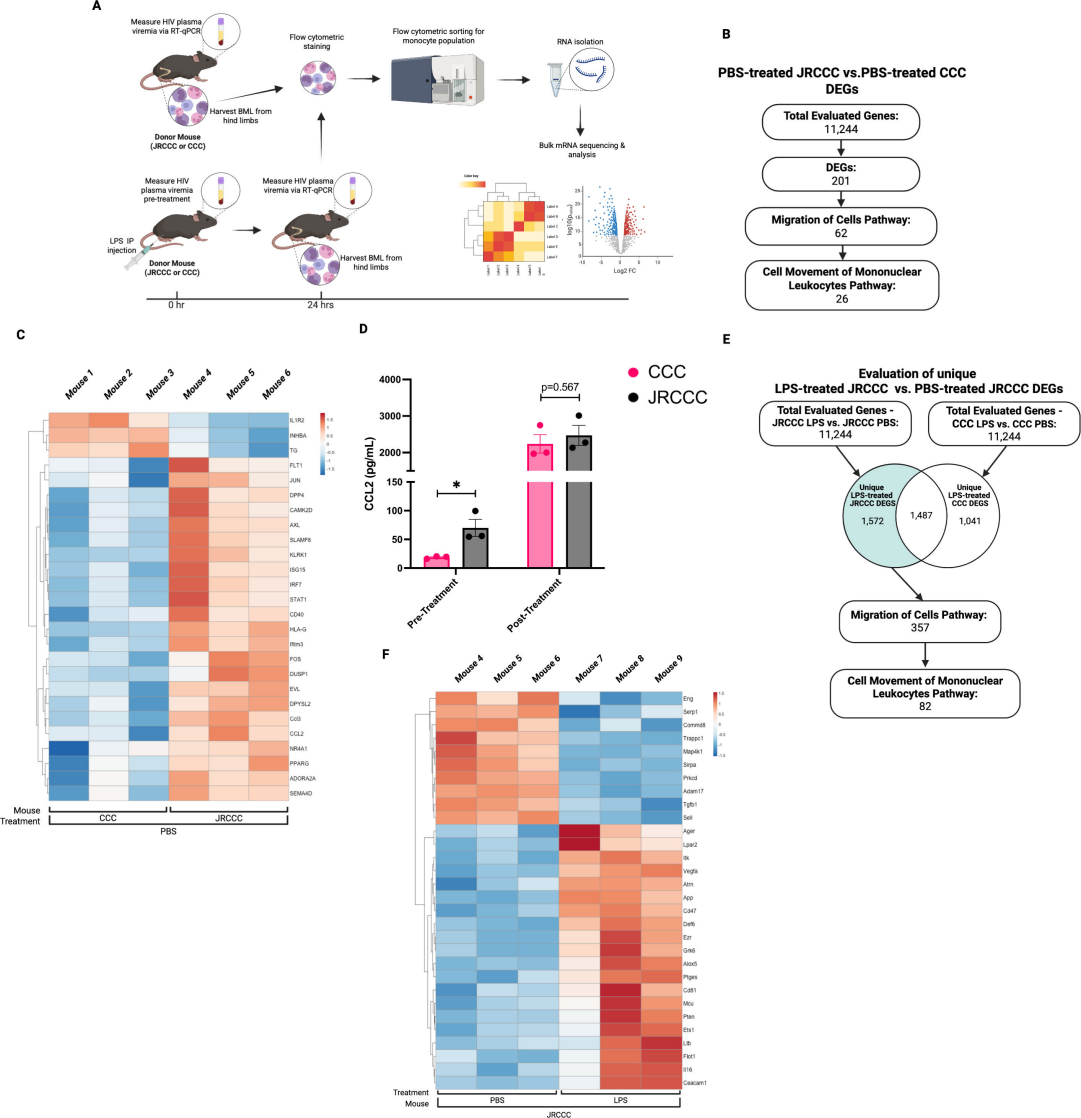
Bulk mRNA sequencing of highly purified monocytes from PBS or LPS-treated JRCCC and CCC mice. (**A**) Highly purified CD11b+ monocytes were isolated by flow cytometric sorting from PBS or LPS-treated (3 mg/kg) JRCCC and CCC mice (*n* = 3 mice per group). RNA was extracted, and gene expression was evaluated by mRNAseq. (**B**) Flow chart of the identification of DEGs in JRCCC PBS vs CCC PBS using an FC >1.5 and *P* <0.01. (**C**) Heatmap of the DEGs associated with the “cell movement of mononuclear leukocytes” pathway for JRCCC PBS vs CCC PBS. The scale bar is the log_2_ FC ratio varying from red (upregulated genes) to blue (downregulated genes). (**D**) Enzyme-linked immunosorbent assay (ELISA) results showing CCL2 secretion levels in plasma of JRCCC and CCC mice (*n* = 3 mice per genotype) pre- and 4 h post-LPS treatment. The bar graph displays the average chemokine concentration of two assays for each mouse with mean ± SEM. Statistical analysis was performed using an unpaired *t*-test between groups (*P ≤ 0.05). (**E**) Flow chart of genes in JRCCC LPS vs JRCCC PBS. DEGs were identified with an FC >1.5 and *P* <0.01. Genes uniquely associated with JRCCC LPS were further evaluated using Ingenuity pathway analysis (IPA). (**F**) Heatmap of the DEGs associated with “cell movement of mononuclear leukocytes” pathway for JRCCC LPS vs JRCCC PBS as shown above (**C**).

We did not observe increased migration of CCC BML across the BBB of LPS-treated B6 recipient mice; therefore, it is unlikely that the increased migration of the JRCCC monocytes into the brains of the LPS-treated B6 recipient mice was primarily due to LPS-mediated compromise of BBB integrity alone. We postulated that LPS treatment increased entry of JRCCC monocytes into the brains of mice by stimulating HIV gene expression in the monocytes. This was supported by the higher viral loads observed in LPS-treated B6 mice compared to PBS-treated controls, in mice injected with highly purified JRCCC monocytes or with BML (Fig. 2E and 3B). Additionally, increasing HIV gene expression induced by LPS treatment likely modulated the expression of cellular genes, which in turn increased the capacity of the JRCCC BML to migrate across the BBB into the brain. Consequently, we investigated potential mechanisms by which HIV gene expression increased the *in vivo* migration of JRCCC BMLs across the BBB by comparing the transcriptomes of highly purified monocytes isolated from PBS or LPS-treated JRCCC and CCC mice. Monocytes were highly purified (~98% CD11b+) from JRCCC or CCC BML (*n* = 3 mice per group) by flow cytometric sorting. RNA was extracted and the monocyte transcriptomes were evaluated by bulk mRNA sequence analysis (Fig. 4A). Differentially expressed genes (DEG) with a fold change (FC) of >1.5 (*P* < 0.01) were identified after comparison of the transcriptomes of monocytes from PBS-treated JRCCC mice vs PBS-treated CCC mice, LPS-treated JRCCC mice vs PBS-treated JRCCC mice, and LPS-treated CCC mice vs PBS-treated CCC mice.

To identify genes associated with the increased capacity of JRCCC monocytes to cross the BBB in the absence of exogenous activation, we compared the transcriptomes of monocytes from PBS-treated JRCCC mice vs PBS-treated CCC mice using Ingenuity Pathway Analysis (IPA). RSAD2/Viperin was identified as a gene upregulated by HIV infection of monocyte-derived macrophages but not by HIV infection of CD4+ T cells and which facilitated the sustained HIV infection of monocyte-derived macrophages (37). Out of a total of 201 DEGs, 62 DEGs are categorized in a “migration of cells” pathway, including 26 DEGs further associated with a “cell movement of mononuclear leukocytes” sub-pathway (Fig. 4B). Heatmaps of the DEGs associated with the cell movement of mononuclear leukocytes from three biological replicates of PBS-treated CCC or JRCCC mice are shown (Fig. 4C). Several of the 26 DEGs had functions reported to be strongly associated with migration and adhesion of monocytes (Table S1). Specifically, there were several genes directly associated with chemotaxis of monocytes, such as *CCL3 (3.23 FC), CCL2 (2.67 FC),* and *FLT1* (*1.75 FC*), and several transcription factors associated with inducing cellular migration, including *FOS* (7.26 *FC*) and *JUN* (2.19 FC). The same 26 DEGs associated with the “cell movement of mononuclear leukocytes” sub-pathway were also assessed in the transcriptomic comparison between LPS-treated JRCCC mice and LPS-treated CCC mice (Table S2). Heatmaps of these DEGs were generated and show that 12 out of 26 genes were significantly different in both the PBS-treated JRCCC vs PBS-treated CCC and LPS-treated JRCCC vs LPS-treated CCC comparisons (Fig. S1). CCL2 is of particular interest because it has been found to mediate monocyte migration across the BBB (38), and levels of CCL2 (MCP-1) were elevated in the cerebrospinal fluid of PWH with human immunodeficiency virus-associated dementia and correlated with dementia severity (39). We measured plasma levels of CCL2 in JRCCC and CCC mice before and 4 h after LPS treatment (3 mg/kg) using an enzyme-linked immunosorbent assay (ELISA) (Fig. 4D). Significantly higher (*P* < 0.05) levels of CCL2 were detected in plasma of untreated JRCCC mice (mean = 70.11 pg/mL) compared to levels observed in untreated CCC mice (mean = 18.88 pg/mL), indicating that HIV production by the JRCCC monocytes was associated with increased CCL2 production. Interestingly, LPS treatment markedly increased CCL2 production in both JRCCC and CCC mice to similar levels, perhaps because it induces maximal CCL2 production. An additional DEG identified relevant to increasing the migratory capacity of JRCCC monocytes across the BBB was *ADORA2A* (2.08 FC). Chronic activation of adenosine receptor 2a (Adora2a)-mediated signaling compromises the BBB by eroding the tight junctions between the endothelial cells of the cerebral vasculature (40). This transcriptome analysis suggested that expression of the HIV provirus in the JRCCC monocytes may induce expression of genes associated with the migration of monocytes, thereby increasing their *in vivo* migration across the BBB compared to CCC monocytes in PBS-treated mice.

Migration of JRCCC mouse BMLs across the BBB in LPS-treated B6 mice was significantly increased compared to their migration across the BBB of PBS-treated B6 mice. In contrast to CCC mouse BML, which displayed equivalent migration across the BBB of either PBS-treated or LPS-treated B6 mice. Therefore, DEGs were identified by comparing monocytes from LPS-treated JRCCC mice vs PBS-treated JRCCC mice (3,059 DEGs) to the DEGs from LPS-treated CCC mice vs PBS-treated CCC mice (2,528 DEGs). This allowed us to identify DEGs unique to monocytes from LPS-treated JRCCC mice (1,572 DEGs) (Fig. 4E). Analysis of these DEGs was assessed by IPA, and 357 DEGs related to a “migration of cells” pathway were identified (Table S3) and included 82 DEGs in the “cell movement of mononuclear leukocytes” sub-pathway. A heatmap of selected DEGs associated with the “cell movement of mononuclear leukocytes” pathway from three biological replicates of PBS and LPS-treated JRCCC mice is shown (Fig. 4F). Several of the observed genes were associated with the migration of monocytes, including *AGER* (*3.35 FC*), *CECAM1* (*2.17 FC*), *VEGFA* (*1.88 FC*), *CD47* (*1.70 FC*), *APP* (*1.63 FC*), *TGFB1* (*−1.63 FC*), and *SIRPA1* (−1.58 FC). In addition, *LPAR2* (1.68 FC) encodes a receptor for lysophosphatidic acid, a bioactive lysophospholipid ligand, which favors interactions between endothelial cells and circulating monocytes (41). Furthermore, *ITK* (2.85 FC) and *DEF6* (1.57 FC) are signaling molecules that play an important role in the trafficking and infiltration of T cells into the CNS and the development and pathogenesis of experimental autoimmune encephalomyelitis (42, 43).

### Effect of morphine or methamphetamine treatment on the *in vivo* migration of JRCCC BML across the BBB

**Fig 5:**
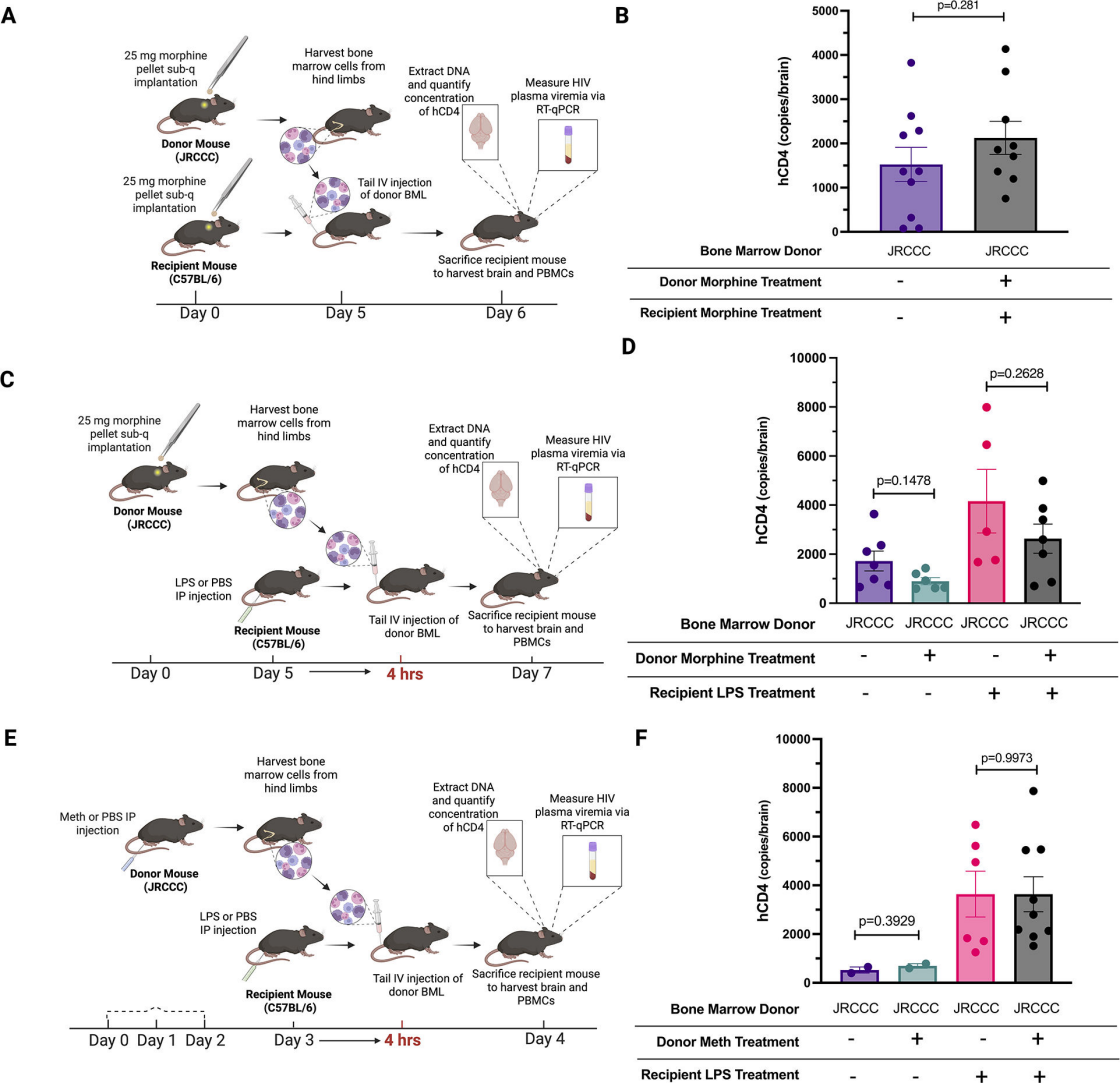
Effect of morphine or methamphetamine on migration of JRCCC BML into the brains of recipient mice. (**A**) Experimental set-up and timeline for donor and recipient mouse morphine treatment. (**B**) Five days post-implantation with morphine (25 mg) or sham pellets, BML from JRCCC mice was harvested and intravenously injected into morphine- or sham-treated B6 recipient mice, respectively. (**C**) Experimental set-up and timeline for donor morphine treatment. (**D**) BML was harvested from JRCCC mice 5 days after implantation with morphine (25 mg) or sham pellets and intravenously injected into B6 mice 4 h after they were treated with LPS (3 mg/kg, *n* = 12 mice) or PBS (*n* = 13 mice). (**E**) Experimental set-up and timeline for methamphetamine treatment. (**F**) BMLs were harvested from JRCCC mice after treatment with escalating doses of methamphetamine (1–5 mg/kg) or PBS for 3 days and intravenously injected into B6 mice 4 h after treatment with either LPS (*n* = 15 mice) or PBS (*n* = 4 mice). For panels B, D, and F, pooled data from independent experiments are shown with each symbol representing an individual mouse and shown as bar graphs with mean ± SEM of human CD4 DNA copies/brain and statistical analysis performed using unpaired *t*-tests between groups.

Divergent results have been reported regarding the impact of the chronic use of drugs of abuse, such as opioids and methamphetamine, by PWH on accelerating cognitive decline. Some studies reported an association between SUD and increased cognitive decline, while others have not observed a significant impact of chronic substance abuse on neurocognitive function (44). Opioids, such as heroin or morphine, can exacerbate and accelerate the onset of compromised cognitive function in PWH (45). The high prevalence of methamphetamine use among PWH has been associated with accelerated progression to AIDS and increased neurocognitive impairment (46, 47). We investigated the effect of morphine on both the migration across the BBB by monocytes capable of producing HIV and on the integrity of the BBB using our JRCCC mouse monocyte adoptive transfer model treated with morphine pellets as described in previous studies of the physiological and organ impacts of morphine treatment (48, 49). We implanted both the donor JRCCC and recipient B6 mice with either a sham or a morphine pellet (25 mg), which provides sustained and continuous systemic delivery of morphine (48, 49). Five days later, the morphine-treated B6 mice were intravenously injected with BML isolated from the morphine-treated JRCCC mouse, and the sham-treated B6 mice were intravenously injected with sham-treated JRCCC mouse BML. One day later, the mice were sacrificed, DNA was extracted from their brains and the number of JRCCC mouse BMLs which migrated into the brains was quantified by measuring the human CD4 copy number by ddPCR (Fig. 5A). The mean human CD4 copy number in the brains of sham-treated B6 mice injected with sham-treated JRCCC mouse BML (1,525 hCD4 copies/brain) was not significantly different (*P* = 0.281) from the mean human CD4 copy number (2,125 hCD4 copies/brain) in the brains of morphine-treated B6 mice injected with morphine-treated JRCCC mouse BML (Fig. 5B). These results indicate that short-term morphine treatment of both the JRCCC monocytes and recipient B6 mouse does not significantly affect migration of monocytes supporting HIV production across the BBB and the permeability of the BBB.

We next examined how treatment of peripheral HIV-producing monocytes with morphine alone affected their migration into the brains of B6 mice with either an intact or LPS-compromised BBB. Five days after implanting morphine or sham pellets into JRCCC mice, their BMLs were harvested and intravenously injected (20 × 10^6^ cells) into PBS or LPS-treated B6 mice (Fig. 5C). Two days later, human CD4 copy numbers in the mouse brains were quantified by ddPCR to determine the number of JRCCC BML cells that migrated into the brain (Fig. 5D). While ddPCR quantification indicated that almost 50% fewer morphine-treated JRCCC mouse BML (mean = 899 ± 142 copies/brain) than sham-treated JRCCC mouse BML (mean = 1,720 ± 405 copies/brain) migrated into the brains of the PBS-treated mice, it was not statistically significant (*P* = 0.148). Similarly, while about 36% fewer morphine-treated JRCCC mouse BML cells (mean = 2,632 ± 596 copies/brain) migrated into the brains of LPS-treated B6 mice than sham-treated mouse JRCCC BML (mean = 4,160 ± 1,293 copies/brain), it was not statistically significant (*P* = 0.263). These results suggest that while there was a trend that short-term morphine treatment of BML alone reduced the migration of HIV-infected BML across the BBB, it was not statistically significant.

To assess whether methamphetamine influences the migration of circulating HIV-producing monocytes across the BBB, we treated JRCCC mice with a methamphetamine regimen that recapitulates methamphetamine binge-use patterns observed in individuals who rapidly escalate dose and frequency over several consecutive days (50). This regimen is a modified version of a previously described methamphetamine dosing strategy in mice (51). JRCCC mice were either treated with PBS or treated with a dose escalation regimen of 4 doses/day of increasing methamphetamine concentrations over 3 days culminating with 4 doses of 5 mg/kg on day 3. On day 4, JRCCC mouse BMLs were harvested from the PBS- and methamphetamine-treated mice and intravenously injected (20 × 10^6^ cells) into either PBS or LPS-treated B6 mice. Two days later, the number of JRCCC mouse BMLs that migrated into the brain was quantified by measuring human CD4 copy number by ddPCR (Fig. 5E). Based on quantification of human CD4 copy number in the brains of the injected PBS-treated B6 mice (Fig. 5F), the mean number of methamphetamine-treated JRCCC mouse BML (mean = 694 ± 94 copies/brain) was comparable to the mean number of PBS-treated JRCCC mouse BML (mean = 525 ± 125 copies/brain). Similarly, in the brains of the injected LPS-treated B6 mice, the mean number of methamphetamine-treated JRCCC mouse BML (mean = 3,636 ± 719 copies/brain) was comparable to the mean number of PBS-treated JRCCC mouse BML (mean = 3,640 ± 938 copies/brain). These results indicated that a short course of methamphetamine does not have a significant effect on the migration across the BBB of HIV-producing BML.

## DISCUSSION

Although antiretroviral therapy (ART) has markedly reduced the severity of HAND, PWH continue to develop milder forms of neurological and cognitive impairment for which no treatment is currently available (52–54). HIV infection is introduced into the brain early in disease progression through the migration of HIV-infected monocytes across the BBB. This process persists throughout the course of the disease, continually replenishing and expanding the HIV reservoir in the brain and driving chronic neuroinflammation, leading to neuronal damage and contributing to neurocognitive impairment (7, 55–57). Identifying mechanisms by which circulating HIV-infected monocytes migrate across the BBB into the brain and contribute to the neuropathogenesis of HAND may enable the development of new treatments. Two factors likely act in tandem to increase the capacity of HIV-infected monocytes to migrate into the brain: compromise of BBB integrity by HIV-induced inflammation and HIV-stimulated changes in monocyte gene expression that increase their migratory capacity across the compromised BBB into the brain (58).

The BBB protects the brain from the deleterious effects of toxic substances and inflammatory cells circulating in the blood by providing a barrier to prevent the passage of most circulating macromolecules and cells into the brain (59). The BBB is composed of junctional complexes formed by the interaction of endothelial cells (ECs), astroglia, and pericytes (60). BBB ECs differ from tissue ECs due to their expression of inter-endothelial tight junctions, which prevent the passage of most molecules and cells across the paracellular pathways between adjacent ECs (59–61). These tight junctions are comprised of three integral membrane proteins: claudin, occludin, and junction adhesion molecules, as well as several cytoplasmic accessory proteins, including Zonula occludens-1, -2, and -3 (30). BBB integrity is commonly evaluated by quantifying the passage of substances normally excluded from the brain by BBB tight junctions, such as Evans blue dye, dextran, sucrose, horseradish peroxidase, or sodium fluorescein (30). Increased passage of low molecular markers, such as sucrose, can reveal even minor reductions in BBB integrity, while higher molecular weight markers, such as dextran, require more extensive compromise of BBB integrity to traverse it (30). Compromise of the BBB, allowing circulating inflammatory cells, such as monocytes, to enter the brain, is a complex process that involves both alterations in circulating leukocytes and a reduction in the tight junction integrity of BBB endothelial cells. These alterations include increased expression of adhesion molecules, such as ICAM-1 and LFA-1, and dysregulated expression of membrane glycoproteins, chemokines, cytokines, matrix metalloproteins, and kinin receptors on circulating leukocytes, combined with increased expression of their ligands, such as VCAM-1 and α4 integrin, on BBB ECs and other cells (61). Consequently, *in vivo* assays using substances, such as dextran or sucrose, are insufficient for evaluating the additional alterations required for inflammatory cells to traverse the BBB and promote HIV infection in the brain.

Given the limitations in monitoring the *in vivo* migration of HIV-infected monocytes across the BBB in PWH, we sought to develop a novel *in vivo* mouse model assay to investigate the mechanisms by which HIV production enhances the capacity of monocytes to migrate across the BBB. By quantifying the entry of circulating HIV-producing monocytes into the brain parenchyma, this model enables investigation of how HIV alone and in combination with other factors, such as LPS and substances of abuse, modulates monocyte transmigration across the BBB and the neuroinflammatory responses that contribute to HAND. To provide a source of primary mouse monocytes displaying active HIV production, we used a transgenic mouse line (JRCCC) constructed to circumvent HIV replication blocks in mice by expressing as tightly linked transgenes a full-length LTR-regulated HIV provirus and CD4 promoter-regulated human CD4, CCR5, and cyclin T1. The presence of these transgenes allowed us to use ddPCR to quantify the number of JRCCC or CCC monocytes or leukocytes infused into B6 mice, which migrated across the intact or LPS-compromised BBB. To measure the effect of HIV Tat, morphine, and *Streptococcus pneumoniae* on BBB integrity, a previous study passively transferred splenocytes from FVB/N mice transgenic for the expression of luciferase under the β-actin promoter into B6CBAF1 mice and quantified leukocytes translocated into the brain by *in vivo* bioluminescence imaging and measurement of luciferase activity in brain lysates (62). The system we developed has two major advantages over that approach for studying the effect of HIV production on BBB integrity. First, it uses monocytes from JRCCC mice that produce HIV and thereby recapitulate the behavior of HIV-infected monocytes crossing the BBB. Second, highly sensitive and quantitative ddPCR specific for the human CD4 or HIV-Gag transgene permitted us to quantify the number of cells that cross the BBB into the brain.

JRCCC monocytes infused into B6 mice displayed active HIV production after injection, as indicated by the presence of HIV plasma viremia, at levels comparable to those observed in untreated PWH (Fig. 2E and 3B). Significantly more JRCCC mouse BML compared to CCC BML crossed the BBB after intravenous injection into PBS or LPS-treated B6 mice (Fig. 3C). Importantly, the selective increased migration of JRCCC mouse BML, but not CCC mouse BML, into the brains of LPS-treated B6 mice indicated that LPS activation of the JRCCC mouse BML HIV-proviral transgenes, rather than compromise of the BBB alone, was responsible for the increased migration of JRCCC mouse BML into the brain. Activation of the JRCCC mouse BML HIV-proviral transgene by LPS treatment of the recipient B6 mice was demonstrated by the almost sevenfold increase in plasma HIV levels in LPS-treated recipient B6 mice intravenously injected with JRCCC mouse BML (mean = 115,653) compared to untreated mice (mean = 16,533) (Fig. 3B). HIV production in HIV-infected monocytes has been reported to activate cellular genes, such as interferon-stimulated genes and neighboring host genes, by local changes in chromatin organization (63–65). Consequently, it is likely that HIV provirus activation by the LPS modulated the downstream expression of genes encoding chemokines, receptors, adhesion molecules, and other factors, which combined to increase the migration of JRCCC monocytes across the BBB. Taken together, these data indicated that expression of HIV, particularly in the presence of LPS, significantly increased the capacity of monocytes to cross the BBB.

LPS, a constituent of the cell walls of gram-negative bacteria, is an endotoxin that can disrupt the BBB and compromise several of its functions, including adsorptive transcytosis, immune cell trafficking, and various transport functions (66). Even with ART, PWH display HIV-related systemic immune activation, associated with increased levels of LPS and other endotoxins in the blood (63), due to compromised gut barrier integrity enabling microbial translocation from the gut into the circulation (66–68). Circulating LPS and other endotoxins may compromise the integrity of the BBB in PWH, thereby facilitating the migration of circulating HIV-infected monocytes (25) and soluble gp120 into the brain (28, 69), which can further compromise BBB integrity and enhance the migration of circulating monocytes into the brain (70). Consequently, the expression of gp120 on the surface of HIV-producing JRCCC monocytes may synergize with LPS to enhance the capacity of monocytes from JRCCC mice, but not CCC mice, to cross the BBB. An intriguing possibility is that broadly neutralizing antibodies directed at gp120 may bind to gp120 and thereby block its deleterious effect on the BBB and reduce the passage of HIV-infected monocytes across the BBB.

We investigated the mechanisms by which JRCCC monocytes displayed increased transmigration across the BBB by performing bulk mRNAseq of highly purified monocytes from mouse bone marrow, followed by transcriptome analysis. Since monocytes from PBS-treated JRCCC mice displayed significantly more migration across the BBB than CCC BMLs, we compared the transcriptomes of PBS-treated JRCCC mouse monocytes vs CCC mouse monocytes and identified 26 DEGs associated with the “cell movement of mononuclear leukocytes” pathway from IPA (Fig. 4B and C). While several of these DEGs were associated with chemotaxis of monocytes, one of them, *CCL2* (C-C motif chemokine ligand 2), plays a key role in the infiltration of monocytes into the CNS of mice associated with production of *MCP-1* and *CCL2* by activated and HIV-infected microglia (71, 72). The upregulated expression of *CCL2* by JRCCC monocytes may contribute to their increased migration across the BBB compared to monocytes from PBS-treated CCC mice. Another upregulated DEG, *ADORA2A*, has been reported to contribute to BBB breakdown, which could synergize with chemokines and other factors to facilitate migration of HIV-infected monocytes across the BBB.

The most robust migration across the BBB occurred after JRCCC mouse monocytes were intravenously injected into LPS-treated B6 mice, which would expose the transferred JRCCC mouse monocytes to LPS and the inflammatory milieu it induces, activating transcription of their LTR-regulated HIV transgenes. We compared the transcriptomes of LPS-stimulated JRCCC vs CCC mouse monocytes to investigate potential mechanisms underlying the enhanced migration of monocytes from LPS-treated JRCCC mice compared to monocytes from LPS-treated CCC mice or PBS-treated JRCCC mice. One DEG we identified, *CEACAM1,* is expressed by monocytes and inhibits monocyte apoptosis and *CEACAM1*-dependent binding interactions (73), which may facilitate monocyte recruitment to inflammatory sites, thereby contributing to the capacity of monocytes to serve as an HIV reservoir in the brain.

Previous studies have shown that *ITK* (interleukin-2-inducible T cell kinase) and *DEF6 (*DEF6 guanine nucleotide exchange factor), both DEGs that were identified in our study, are genes associated with cellular infiltration to the CNS (42, 43). The knockout of *ITK* in mice is associated with reduced migration of CD4 T cells and increased disease severity in an autoimmune encephalomyelitis mouse model (43), suggesting that upregulation of *ITK* in monocytes may increase their capacity to migrate from the peripheral blood into the brain. Another DEG we identified, *DEF6*, was upregulated in the monocytes from LPS-treated JRCCC mice. Because downregulation of *DEF6* decreases the infiltration of cells into the CNS (42), upregulation of *DEF6* may increase the capacity of monocytes to migrate across the BBB. These are examples of upregulated genes that modulate monocyte migration and may contribute to the increased capacity of HIV-infected monocytes to cross the BBB into the brain in the presence of LPS and other endotoxins in the plasma of PWH. Further analysis by correlating the expression of these and other DEGs identified in our study in monocytes from PLWH may provide further insights into the neuropathogenesis of HAND and identify potential therapeutic targets to prevent or ameliorate HAND.

We used this model to study the impact of substances of abuse, such as morphine and methamphetamine, on the migration of JRCCC BML across the BBB. While not statistically significant, our results suggest that morphine treatment reduces the migration of JRCCC BML across the BBB into the brains of PBS or LPS-treated mice. Differing results were reported in previous studies investigating the impact of morphine treatment on BBB integrity using various *in vitro* and *in vivo* assays. One *in vitro* study reported that morphine use may increase cell migration into the CNS based on their observation that prolonged *in vitro* exposure of a human brain microvascular endothelial cell line to morphine increased their surface expression of *ICAM-1, VCAM-*1, and *ALCAM* and the binding of human CD3+ PBMCs (74). Another *in vitro* study using brain microvascular endothelial cells reported that morphine treatment reduced the expression of tight junction proteins ZO-1, occludin, and *JAM-2* and enhanced migration of HIV-infected PBMC across an *in vitro* BBB model (75). Our results are similar to an *in vivo* study, which reported that treatment of rats daily for 12 days with intraperitoneal injection of morphine did not reduce BBB integrity as measured by Evans blue or ^[131]^ iodine tracer extravasation in the thalamus, hypothalamus, or cerebral cortex (76, 77). Similarly, an *in vivo* study that adoptively transferred luciferase-expressing leukocytes from transgenic mice into recipient mice implanted with morphine pellets (25 mg) did not detect any effect of morphine treatment on the trafficking of inflammatory monocytes or other cells across the BBB into the brain (62).

Our studies did not detect any effect of short-term methamphetamine treatment on the passage of circulating JRCCC BML across the BBB into the brain. While one study reported that methamphetamine treatment of mice disrupted the integrity of their BBB, manifested by profound leakage of albumin in the cerebral cortex, this occurred 4 h after the administration of a very high methamphetamine dose (40 mg/kg), which is far higher than the doses used by methamphetamine users (77, 78) or used in this study.

Taken together, our results demonstrate that expression of HIV increases the *in vivo* capacity of monocytes to cross the BBB, particularly in the presence of LPS, and identify multiple DEGs that may play a role in enabling HIV-infected monocytes to cross the BBB. Furthermore, it describes a novel *in vivo* model that can be used to study the impact of HIV infection alone and in combination with other factors, such as substances of abuse, on BBB integrity and the migration of HIV-infected monocytes across the BBB, and the efficacy of new therapies to inhibit further migration of HIV-infected monocytes across the BBB.

## MATERIALS AND METHODS

### Construction of transgenic mice

A vector expressing CD4 promoter/enhancer-regulated human CD4 and CCR5 as a single transcript linked by a self-cleaving picornavirus-derived 2A peptide sequence (P2A) and a construct expressing CD4 promoter/enhancer-regulated human cyclin T1 were constructed as previously described (23). An infectious molecular clone of HIV-1_JR-CSF_, PYK-JR-CSF, isolated from an HIV-infected individual (79) was obtained from the NIH HIV Reagent Program (Division of AIDS, NIAID, NIH, Rockville, MD). We generated mice transgenic for the JR-CSF provirus regulated by its endogenous LTR and human CD4, CCR5, and cyclin T1 regulated by the CD4 promoter/enhancer cassette as tightly linked transgenes (JRCCC mice) by microinjecting the human CD4-P2A-CCR5, the human cyclin T1, and the HIV_JR-CSF_ constructs together into fertilized oocytes from C57BL/6 mice as we previously described (22, 23). Mice transgenic for human CD4, CCR5, and cyclin T1 regulated by the CD4 promoter/enhancer cassette (CCC mice) were generated as described (23).

### Western blot

Expression of human CCR5 and cyclin T1 in the CCC mouse CD4 T cells was detected by western blot analysis of splenocytes from the CCC mice, with splenocytes from B6 mice serving as a negative control and MOLT4 cells, which express human CCR5 (80) (NIH HIV Reagent Program) as a positive control. After harvesting the cells, they were lysed in RIPA buffer with added protease inhibitor cocktail (complete tablet, Roche, Basel, Switzerland) using 100 µL/10 × 10^6^ cells by incubation on ice for 1 h while vortexing every 10 min. After the cell lysates were centrifuged at 14,000 rpm for 30 min at 4°C, the supernatant was collected and the protein concentration was quantified via Pierce BCA protein concentration assay (ThermoFisher, Waltham, MA). Samples (20 µg) were heated at 95°C for 5 min with an equal volume of 2× Laemmli Buffer (Bio-Rad, Hercules, CA) and size fractionated on a 4%–20% Mini PROTEAM TGX Precast Protein Gel (Bio-Rad) at 150 V for 45 min. After transferring the fractionated proteins onto nitrocellulose membranes using the iBlot 2 gel transfer device and iBlot Transfer Stack, NC (Invitrogen), the membranes were incubated with blocking buffer (PBS with added 5% non-fat dry powdered milk and 0.1% Tween) for 1 h at room temperature on a shaker. The membranes were then washed in PBS with added 0.1% Tween (PBS-T) and incubated for 1.5 h at room temperature on a shaker with either rabbit polyclonal anti-β-actin, 1:2,000 (20536-1-AP, Proteintech, Chicago, IL), rabbit polyclonal anti-human CCR5, 1:500 (174761AP, Proteintech), or goat polyclonal anti-human Cyclin-T1, 1:1,000 (sc-8127, Santa Cruz Biotechnology, Dallas, TX), washed again in PBS-T and then incubated for 1 h at room temperature on a shaker with the relevant secondary polyclonal antibody conjugated to horseradish peroxidase (mouse polyclonal anti-rabbit IgG 1:1,000 (93702S, Cell Signaling Technology, Danvers, MA) or rabbit anti-goat IgG (H+L) 1:1,000 (6160-05, Southern Biotech, Birmingham, AL). After the membranes were washed with 1× PBS-T, they were incubated with the Immobilon Western HRP Substrate (WBLKS0100, Millipore Sigma, Burlington, MA), and antibody-binding proteins were visualized using the ChemiDoc Touch Imaging system (Bio-Rad) and analyzed using the Image Lab program (Bio-Rad).

### Isolation of mouse bone marrow leukocytes containing bone-marrow-derived monocytes

BML were isolated from mouse femurs and tibias by flushing with PBS and separated from bone shards by passing the suspension through a 70 µM cell strainer. Red blood cells were lysed using 1× RBC lysis buffer, and the remaining cells were washed and resuspended in sterile PBS. In some experiments, highly purified CD11b+ monocytes (>90% CD11b+) were isolated from the bone marrow leukocytes after 1× RBC lysis by immunomagnetic separation using CD11b mouse MicroBeads and a MiniMACS Separator column (Miltenyi Biotec, Gaithersburg, MD) and then washed and resuspended in PBS as described (81).

### Evaluation of *in vivo* migration of bone marrow leukocytes across the blood-brain barrier

Prior to the intravenous injection of BML from donor JRCCC or CCC mice, recipient C57BL/6 mice (B6 mice) were either intraperitoneally injected with LPS (3 mg/kg) from *Escherichia coli* O55:B5 (Sigma-Aldrich, Saint Louis, MO) to increase the permeability of the BBB or with PBS as a negative control. Four hours later, recipient B6 mice were anesthetized with xylazine/ketamine (10 mg/kg of body weight), and BML (~20 × 10^6^ cells) from JRCCC or CCC transgenic donor mice were injected intravenously into the tail veins. Two days later, recipient B6 mice were anesthetized, exsanguinated, and intracardially perfused with PBS (30 mL) to clear blood from the brain circulation. Following perfusion, the whole brain was harvested, placed in Hanks’ Balanced Salt Solution (HBSS) (Corning), and minced using a razor blade. The minced brain in HBSS is transferred to a 15 mL conical and spun for 2 min at 1,200 rpm. After aspiration, single-cell suspensions of whole brain were obtained using the Neural Tissue Dissociation Kit (P) (Miltenyi Biotec), and DNA was extracted from whole brain cell lysate using the AllPrep DNA/RNA kit (QIAGEN) according to the manufacturer’s protocols and resuspended in EB Buffer (100 µL).

### Quantification of donor cell migration into the brains of recipient mice by droplet digital PCR

We leveraged the presence of the human CD4 and HIV provirus transgenes in the JRCCC or CCC donor mouse BML we injected to use ddPCR to quantify the number of donor cells that traversed the BBB into the brain by determining the copy number of the human CD4 or HIV-Gag sequences in the recipient mouse brains. DNA was extracted from whole brain cell lysate using the AllPrep DNA/RNA Kit (QIAGEN) according to the manufacturer’s instructions, resuspended in EB Buffer (100 µL), and the DNA concentration was determined using a Nanodrop 2000 Spectrophotometer (ThermoFisher). The number of copies of human CD4 and HIV-Gag DNA in brain cell lysate DNA (25 ng/µL) was quantified by ddPCR using PrimePCR Custom Assay human-specific CD4 and HIV-Gag primer pairs and probe mixes (Bio-Rad, Hercules, California). The human CD4 primer/probe mix sequences used were hCD4 forward primer (5′-GGCCTCCAGCATAGTCTATAAG-3′), hCD4 reverse primer (5′-AGGTCAAAGGTGATCCAAGAC-3′), and hCD4 probe (5′-GTTCTCCTTCCCACTCGCCTTTACA-3′) with dye quencher (5′ 6-FAM, 3′ Iowa Black FQ). We also used HIV-Gag primer/probe mix sequences specific for highly conserved HIV-*Gag3* regions, HIV-Gag forward primer (5′-TCAGCCCAGAAGTAATACCCATGT-3′), reverse primer (5′-CACTGTGTTTAGCATGGTGTTT-3′), and probe (5′-ATTATCAGAAGGAGCCACCCCACAAGA-3′) with dye quencher (5′ 6-FAM, 3′ Iowa Black FQ). DNA extracted from splenocytes of our JRCCC transgenic mouse carrying the human CD4 transgene was diluted in nuclease-free water (1:10,000) and used as a positive control. Similarly, DNA from B6 splenocytes was diluted and used as a negative control.

The ddPCR reaction consisted of brain cell DNA samples (10 µL, 250 ng) and a master mix (11 µL) containing ddPCR Supermix for probes (no dUTP), primers (900 nM), and a FAM-labeled CD4 or HIV-Gag double quencher-probe (200 nM). The sample DNA and master mix were thoroughly mixed, and an aliquot (20 µL) was transferred to an 8-channel DG8 cartridge with oil (70 µL) for droplet generation via the QX200 droplet generator (Bio-Rad). Droplets (40 µL) were transferred to a semi-skirted white/clear 96-well plate (Bio-Rad), which was sealed with a pierceable foil heat seal in the PX1 PCR Plate Sealer (Bio-Rad) and placed into the C1000 Touch Thermal Cycler (Bio-Rad). Amplification was performed by activation of the enzyme at 95°C for 10 min, followed by 40 cycles of 94°C (30 s) and 60°C (1 min), followed by incubation at 98°C (10 min). After cooling the samples to 4°C, the plate was transferred to the QX200 Droplet Reader (Bio-Rad) for analysis of the droplets using the Quantasoft Version 1.6.6 program and the application of manual thresholds.

### ddPCR data analysis

CD4 or HIV-Gag copy number/cell was calculated as [gene copies/(HBB copies/2)]. Intra- and inter-assay coefficients of variability were calculated for method optimization. For descriptive statistics, IBM SPSS Statistics version 22 was used (IBM, New York, NY). ddPCR detection of human CD4 or HIV-Gag in samples was reported in copies/μL determined by QuantaSoft software and compared between treatment groups with all samples normalized to 25 ng/μL of DNA, as recommended per Bio-Rad protocol. CD4 or HIV-Gag copy number/brain was calculated as [(raw copy number/20 µL) (whole brain DNA concentration × 10 µL), previous equation/(initial DNA concentration × 100 µL)]. Statistical analysis was performed using unpaired *t*-tests between groups using GraphPad Prism (v.8.4) with significance set at *P* < 0.05.

### Plasma viral load measurements

The HIV viral load in mouse plasma was quantified by RT-qPCR as previously described (81). After total RNA was extracted from mouse plasma (50 to 200 μL) using a QIAamp MinElute virus spin kit (Qiagen, Germantown, MD), cDNA was generated using a high-capacity RNA-to-cDNA kit (Applied Biosystems, Foster City, CA) and HIV-specific RT-qPCR was performed using TaqMan Universal Master Mix II, no UNG (Applied Biosystems) with added cDNA (2 μL), and the HIV-Gag sequence-specific primers (5′-TCAGCCCAGAAGTAATACCCATGT-3′ [sense] and 5′-CACTGTGTTTAGCATGGTGTTT-3′) and an HIV *gag* sequence-specific (antisense) probe (6FAM-ATTATCAGAAGGAGCCACCCCACAAGA-TAMRA) as described previously (82). Cycle threshold values were calculated using a standard curve and samples with known copy numbers of absolute HIV DNA.

### Evaluation of differential gene expression by bulk mRNAseq

Highly purified CD11b monocytes were isolated from JRCCC or CCC mouse BML by immunomagnetic sorting using CD11b mouse microbeads (Miltenyi Biotec). Monocytes (~1 × 10^6^ cells) were counted and lysed in Buffer RLT Plus. RNA was extracted from the cell lysate using the RNeasy Micro Kit (QIAGEN) according to the manufacturer’s instructions. RNA was resuspended in RNase-free water (14 µL) and its concentration determined using a NanoDrop 2000 Spectrophotometer (ThermoFisher). After RNA integrity and purity were confirmed by analysis on 1% agarose gels, the NanoPhotometer spectrophotometer (Implen, Westlake Village, CA), and the RNA Nano 6000 Assay Kit and Bioanalyzer 2100 system (Agilent, Santa Clara, CA), bulk mRNA sequencing analysis using total RNA from each sample (~20 ng) was performed by Novogene (Durham, NC). Sequencing libraries were generated from the RNA (1 μg/sample) using the NEBNext UltraTM RNA Library Prep Kit for Illumina (NEB, Ipswich, MA) following the manufacturer’s recommendations, and index codes were added to attribute sequences to each sample. In brief, mRNA purified from total RNA using poly-T oligo-attached magnetic beads was fragmented, and double-stranded cDNA synthesis was primed using random hexamers and M-MuLV Reverse Transcriptase (RNase H-) for the first strand and DNA Polymerase I and RNase H for the second strand. Library fragments were purified with the AMPure XP system (Beckman Coulter, Brea, CA) to select for cDNA fragments with an average length of 150–200 bp, which were ligated with adaptors, clustered, and sequenced on an Illumina platform.

### Analysis of bulk mRNAseq data

Raw FASTQ files were checked for their quality using FastQC (v0.11.8) (83). Cutadapt (v2.0) (84) was used to remove short or low-quality reads and adapters. Trimmed reads were then aligned to the mouse genome, mm10, using the STAR aligner (v2.4.2) (85) with default parameters. The alignment results were then evaluated through Qualimap (86) (v.2.2.2a) to confirm that all samples had consistent coverage, alignment rate, and no 5′ or 3′ bias. The Ensembl gene model was used to count and summarize the uniquely mapped reads that overlap with known exons/features through Feature Counts v1.5.0 post3 (87). The edge library (88) was used in gene expression analysis to filter low-expressed reads using CPM and normalize the samples using the TMM method for further analysis. Voom was used to estimate the differential expression among the biological samples from monocytes isolated from three mice for each experimental condition. To lower the type I error rate and increase confidence, a *P* value cutoff of 0.01 (*P* <0.01) was used to select the unique genes with a significant expression change of >1.5-fold.

### Functional enrichment and pathway analysis

Functional analysis of DEG was performed using Ingenuity pathway analysis (IPA; Qiagen). Each gene was mapped to its corresponding molecule in the Ingenuity Pathways Knowledge Base using the Ensembl gene identifier, along with a corresponding *P* value <0.01 and an absolute FC of >1.5. Genes associated with the pathway identified by IPA as “migration of cells” were further characterized with the pathway identified by IPA as “cell movement of mononuclear leukocytes.”

### Determination of systemic cytokine levels in serum following LPS treatment

Blood was sampled from pre-treated and 4 h post-LPS treated (3 mg/kg) JRCCC and CCC mice from the sublingual vein. Serum was prepared from the blood by centrifuging at 8,000 rpm for 8 min, and cytokine levels were determined using mouse DuoSet ELISAs (R&D, #DY479-05, #DY466-05). ELISAs were performed according to the manufacturer’s protocol.

### Morphine treatment

JRCCC and/or B6 mice were treated by implantation of sham or 25 mg sustained-release morphine pellets (obtained from NIDA, Bethesda, MD) by subcutaneous placement between the shoulder blades. After 5 days, the mice were sacrificed, and BML was harvested for subsequent analysis.

### Methamphetamine treatment

A modified protocol was used to treat JRCCC mice with a methamphetamine binge regimen over the course of several days (51). The methamphetamine treatment consisted of intraperitoneal injections of PBS or methamphetamine (methamphetamine hydrochloride; Sigma) every 2 h for 3 days, with a gradual dose escalation giving four intraperitoneal injections/day. On day 1, the mice received two doses of 1 mg/kg, followed by two doses of 2 mg/kg. On day 2, the mice received two doses of 3 mg/kg, followed by two doses of 4 mg/kg. On day 3, the mice received four doses of 5 mg/kg. The following day, mice were sacrificed, and BMLs were harvested for subsequent analysis.

### Evaluation of BBB integrity by immunohistochemistry

Four hours after recipient B6 mice were intraperitoneally injected with LPS (3 mg/kg) or PBS, they were anesthetized and perfused by sequential intracardiac injection of 1× PBS (30 mL) and 4% PFA (25 mL). Brains were harvested and washed in cold PBS (10 mL), followed by immersion in 10% sucrose solution in a 15 mL conical tube until the brains sank to the bottom. Brain tissues were then transferred to a 20% sucrose solution, followed by a 30% sucrose solution; both immersions took place overnight (16 h) at 4°C. Brains were washed three times in cold PBS, bisected coronally, embedded in Tissue-Tek optimal cutting temperature compound (Sakura Finetek, Torrance, CA), and stored at −80°C until sectioning. Frozen, embedded tissues were sectioned with a Leica CM cryostat (Leica Biosystems, Buffalo Grove, IL) at a 15-µm thickness in the coronal plane, collected on Superfrost/Plus microscope slides (Fisher Scientific, #12-550-15), and stored at −80°C until further processing. Tissue sections were submerged in ice-cold acetone for 10 min, followed by three washes (5 min each) in 1× PBS. Tissue sections were then blocked in 0.1 M PBS containing 10% horse serum and 3% Triton X-100 for 1 h at room temperature, followed by overnight incubation at 4°C with the following primary antibodies, claudin-5 polyclonal rabbit antibody (1:550, Thermo Fisher, #34-1600), ZO-1 monoclonal rat antibody (1:300, Thermo Fisher, #14-9776-82), or occludin polyclonal guinea pig antibody (1:50, Origene, #AP26410PU-N). Following primary antibody incubation, tissue sections were washed with PBS three times and incubated with the relevant secondary antibody for 1 h at room temperature, Alexa Fluor 555-labeled goat anti-rabbit IgG (1:600, Thermo Fisher, #A-21428), Alexa Fluor 647-labeled chicken anti-rat IgG (1:1,000, Thermo Fisher, #A-21472), or Alexa Fluor 488-labeled goat anti-guinea pig IgG (1:1,000, Thermo Fisher, #A-11073). Slides were then washed three times in PBS, the brain slice was mounted, and the nuclei were stained with ProLong Gold Antifade Mountant with DAPI (Invitrogen, #P36931) and cover slipped.

### Immunofluorescent image acquisition and ImageJ vessel analysis

Hypothalamic and thalamic regions of six brain tissue sections per mouse were visually evaluated for claudin-5, ZO-1, and occludin staining at 20× magnification using an EVOS M7000 imaging system (Thermo Fisher, #AMF7000). For each treatment condition, TIFF images were acquired of four representative fields from a single brain section, uploaded, and processed using ImageJ software (NIH, Bethesda, MD). The Fiji version of ImageJ containing the plugins Skeletonize (2D/3D) and Analyze Skeleton (2D/3D) was used to analyze BBB vasculature for the merged fluorescent image of each treatment condition. Images were changed to 16-bit (Image → type → 16 bit) and then converted to binary (Process → binary → make binary). Images were then skeletonized using the ImageJ plugin (Process → binary → skeletonize [2D/3D]). To validate the Skeletonize (2D/3D) program, a duplicate image was uploaded to ImageJ and overlaid at 50% transparency (Image → overlay → add image), and invalid branches/vessels were pruned using the selection tool. Average branch length was calculated by the Analyze Skeleton (2D/3D) plugin and obtained from the Results table produced by the program. Statistical analysis was performed using unpaired *t*-tests between treatment groups using GraphPad Prism (v.8.4) with significance set at *P ≤* 0.05.

## Data Availability

All RNA sequencing data are available in GEO under accession no. GSE319558.

## SUPPLEMENTAL MATERIAL

Supplemental material jvi.02063-25-s0001.docx Tables S1 to S3; Fig. S1.

ASM does not own the copyrights to Supplemental Material that may be linked to, or accessed through, an article. The authors have granted ASM a non-exclusive, world-wide license to publish the Supplemental Material files. Please contact the corresponding author directly for reuse.

## References

[1] Burdo TH, Lackner A, Williams KC. 2013. Monocyte/macrophages and their role in HIV neuropathogenesis. Immunol Rev 254:102–113. doi:10.1111/imr.12068 23772617 PMC3704190

[2] Crowe S, Zhu T, Muller WA. 2003. The contribution of monocyte infection and trafficking to viral persistence, and maintenance of the viral reservoir in HIV infection. J Leukoc Biol 74:635–641. doi:10.1189/jlb.0503204 12960232

[3] Fischer-Smith T, Croul S, Sverstiuk AE, Capini C, L’Heureux D, Régulier EG, Richardson MW, Amini S, Morgello S, Khalili K, Rappaport J. 2001. CNS invasion by CD14+/CD16+ peripheral blood-derived monocytes in HIV dementia: perivascular accumulation and reservoir of HIV infection. J Neurovirol 7:528–541. doi:10.1080/135502801753248114 11704885

[4] Valcour V, Chalermchai T, Sailasuta N, Marovich M, Lerdlum S, Suttichom D, Suwanwela NC, Jagodzinski L, Michael N, Spudich S, van Griensven F, de Souza M, Kim J, Ananworanich J, RV254/SEARCH 010 Study Group. 2012. Central nervous system viral invasion and inflammation during acute HIV infection. J Infect Dis 206:275–282. doi:10.1093/infdis/jis326 22551810 PMC3490695

[5] Williams DW, Eugenin EA, Calderon TM, Berman JW. 2012. Monocyte maturation, HIV susceptibility, and transmigration across the blood brain barrier are critical in HIV neuropathogenesis. J Leukoc Biol 91:401–415. doi:10.1189/jlb.0811394 22227964 PMC3289493

[6] Williams DW, Anastos K, Morgello S, Berman JW. 2015. JAM-A and ALCAM are therapeutic targets to inhibit diapedesis across the BBB of CD14+CD16+ monocytes in HIV-infected individuals. J Leukoc Biol 97:401–412. doi:10.1189/jlb.5A0714-347R 25420915 PMC4304417

[7] Williams DW, Calderon TM, Lopez L, Carvallo-Torres L, Gaskill PJ, Eugenin EA, Morgello S, Berman JW. 2013. Mechanisms of HIV entry into the CNS: increased sensitivity of HIV infected CD14+CD16+ monocytes to CCL2 and key roles of CCR2, JAM-A, and ALCAM in diapedesis. PLoS One 8:e69270. doi:10.1371/journal.pone.0069270 23922698 PMC3724935

[8] Ziegler-Heitbrock L. 2007. The CD14+ CD16+ blood monocytes: their role in infection and inflammation. J Leukoc Biol 81:584–592. doi:10.1189/jlb.0806510 17135573

[9] Kallianpur KJ, Valcour VG, Lerdlum S, Busovaca E, Agsalda M, Sithinamsuwan P, Chalermchai T, Fletcher JLK, Tipsuk S, Shikuma CM, Shiramizu BT, Ananworanich J, SEARCH 011 study group. 2014. HIV DNA in CD14+ reservoirs is associated with regional brain atrophy in patients naive to combination antiretroviral therapy. AIDS 28:1619–1624. doi:10.1097/QAD.0000000000000306 25232899 PMC4170526

[10] Kusao I, Shiramizu B, Liang CY, Grove J, Agsalda M, Troelstrup D, Velasco VN, Marshall A, Whitenack N, Shikuma C, Valcour V. 2012. Cognitive performance related to HIV-1-infected monocytes. J Neuropsychiatry Clin Neurosci 24:71–80. doi:10.1176/appi.neuropsych.11050109 22450616 PMC3335340

[11] Shiramizu B, Gartner S, Williams A, Shikuma C, Ratto-Kim S, Watters M, Aguon J, Valcour V. 2005. Circulating proviral HIV DNA and HIV-associated dementia. AIDS 19:45–52. doi:10.1097/00002030-200501030-00005 15627032 PMC1557628

[12] Cysique LA, Hey-Cunningham WJ, Dermody N, Chan P, Brew BJ, Koelsch KK. 2015. Peripheral blood mononuclear cells HIV DNA levels impact intermittently on neurocognition. PLoS One 10:e0120488. doi:10.1371/journal.pone.0120488 25853424 PMC4390276

[13] Valcour VG, Ananworanich J, Agsalda M, Sailasuta N, Chalermchai T, Schuetz A, Shikuma C, Liang C-Y, Jirajariyavej S, Sithinamsuwan P, Tipsuk S, Clifford DB, Paul R, Fletcher JLK, Marovich MA, Slike BM, DeGruttola V, Shiramizu B, SEARCH 011 Protocol Team. 2013. HIV DNA reservoir increases risk for cognitive disorders in cART-naïve patients. PLoS One 8:e70164. doi:10.1371/journal.pone.0070164 23936155 PMC3729685

[14] Rademeyer KM, R Nass S, Jones AM, Ohene-Nyako M, Hauser KF, McRae M. 2024. Fentanyl dysregulates neuroinflammation and disrupts blood-brain barrier integrity in HIV-1 Tat transgenic mice. J Neurovirol 30:1–21. doi:10.1007/s13365-023-01186-4 PMC11232468 38280928

[15] Murphy A, Barbaro J, Martínez-Aguado P, Chilunda V, Jaureguiberry-Bravo M, Berman JW. 2019. The effects of opioids on HIV neuropathogenesis. Front Immunol 10:2445. doi:10.3389/fimmu.2019.02445 31681322 PMC6813247

[16] Chilunda V, Calderon TM, Martinez-Aguado P, Berman JW. 2019. The impact of substance abuse on HIV-mediated neuropathogenesis in the current ART era. Brain Res 1724:146426. doi:10.1016/j.brainres.2019.146426 31473221 PMC6889827

[17] Deng H, Liu R, Ellmeier W, Choe S, Unutmaz D, Burkhart M, Marzio PDi, Marmon S, Sutton RE, Hill CM, Davis CB, Peiper SC, Schall TJ, Littman DR, Landau NR. 1996. Identification of a major co-receptor for primary isolates of HIV-1. Nature 381:661–666. doi:10.1038/381661a0 8649511

[18] Fujinaga K, Taube R, Wimmer J, Cujec TP, Peterlin BM. 1999. Interactions between human cyclin T, Tat, and the transactivation response element (TAR) are disrupted by a cysteine to tyrosine substitution found in mouse cyclin T. Proc Natl Acad Sci USA 96:1285–1290. doi:10.1073/pnas.96.4.1285 9990016 PMC15455

[19] Wei P, Garber ME, Fang SM, Fischer WH, Jones KA. 1998. A novel CDK9-associated C-type cyclin interacts directly with HIV-1 Tat and mediates its high-affinity, loop-specific binding to TAR RNA. Cell 92:451–462. doi:10.1016/s0092-8674(00)80939-3 9491887

[20] Imai K, Asamitsu K, Victoriano AFB, Cueno ME, Fujinaga K, Okamoto T. 2009. Cyclin T1 stabilizes expression levels of HIV-1 Tat in cells. FEBS J 276:7124–7133. doi:10.1111/j.1742-4658.2009.07424.x 20064163

[21] Nixon B, Fakioglu E, Stefanidou M, Wang Y, Dutta M, Goldstein H, Herold BC. 2014. Genital herpes simplex virus type 2 infection in humanized HIV-transgenic mice triggers HIV shedding and is associated with greater neurological disease. J Infect Dis 209:510–522. doi:10.1093/infdis/jit472 23990571 PMC3903370

[22] Sun J, Soos T, Kewalramani VN, Osiecki K, Zheng JH, Falkin L, Santambrogio L, Littman DR, Goldstein H. 2006. CD4-specific transgenic expression of human cyclin T1 markedly increases human immunodeficiency virus type 1 (HIV-1) production by CD4+ T lymphocytes and myeloid cells in mice transgenic for a provirus encoding a monocyte-tropic HIV-1 isolate. J Virol 80:1850–1862. doi:10.1128/JVI.80.4.1850-1862.2006 16439541 PMC1367149

[23] Seay K, Qi X, Zheng JH, Zhang C, Chen K, Dutta M, Deneroff K, Ochsenbauer C, Kappes JC, Littman DR, Goldstein H. 2013. Mice transgenic for CD4-specific human CD4, CCR5 and cyclin T1 expression: a new model for investigating HIV-1 transmission and treatment efficacy. PLoS One 8:e63537. doi:10.1371/journal.pone.0063537 23691059 PMC3655194

[24] Cann AJ, Zack JA, Go AS, Arrigo SJ, Koyanagi Y, Green PL, Koyanagi Y, Pang S, Chen IS. 1990. Human immunodeficiency virus type 1 T-cell tropism is determined by events prior to provirus formation. J Virol 64:4735–4742. doi:10.1128/JVI.64.10.4735-4742.1990 2398528 PMC247960

[25] Jiang W, Luo Z, Stephenson S, Li H, Di Germanio C, Norris PJ, Fuchs D, Zetterberg H, Gisslen M, Price RW. 2021. Cerebrospinal fluid and plasma lipopolysaccharide levels in human immunodeficiency virus type 1 infection and associations with inflammation, blood-brain barrier permeability, and neuronal injury. J Infect Dis 223:1612–1620. doi:10.1093/infdis/jiaa765 33320240 PMC8136977

[26] Kadoki M, Choi BI, Iwakura Y. 2010. The mechanism of LPS-induced HIV type I activation in transgenic mouse macrophages. Int Immunol 22:469–478. doi:10.1093/intimm/dxq032 20504885

[27] Pomerantz RJ, Feinberg MB, Trono D, Baltimore D. 1990. Lipopolysaccharide is a potent monocyte/macrophage-specific stimulator of human immunodeficiency virus type 1 expression. J Exp Med 172:253–261. doi:10.1084/jem.172.1.253 2193097 PMC2188186

[28] Banks WA, Kastin AJ, Brennan JM, Vallance KL. 1999. Adsorptive endocytosis of HIV-1gp120 by blood-brain barrier is enhanced by lipopolysaccharide. Exp Neurol 156:165–171. doi:10.1006/exnr.1998.7011 10192787

[29] Henry CJ, Huang Y, Wynne AM, Godbout JP. 2009. Peripheral lipopolysaccharide (LPS) challenge promotes microglial hyperactivity in aged mice that is associated with exaggerated induction of both pro-inflammatory IL-1beta and anti-inflammatory IL-10 cytokines. Brain Behav Immun 23:309–317. doi:10.1016/j.bbi.2008.09.002 18814846 PMC2692986

[30] Kadry H, Noorani B, Cucullo L. 2020. A blood-brain barrier overview on structure, function, impairment, and biomarkers of integrity. Fluids Barriers CNS 17:69. doi:10.1186/s12987-020-00230-3 33208141 PMC7672931

[31] Sun W, Rassadkina Y, Gao C, Collens SI, Lian X, Solomon IH, Mukerji SS, Yu XG, Lichterfeld M. 2023. Persistence of intact HIV-1 proviruses in the brain during antiretroviral therapy. eLife 12. doi:10.7554/eLife.89837.3 PMC10631759 37938115

[32] Schindelin J, Arganda-Carreras I, Frise E, Kaynig V, Longair M, Pietzsch T, Preibisch S, Rueden C, Saalfeld S, Schmid B, Tinevez JY, White DJ, Hartenstein V, Eliceiri K, Tomancak P, Cardona A. 2012. Fiji: an open-source platform for biological-image analysis. Nat Methods 9:676–682. doi:10.1038/nmeth.2019 22743772 PMC3855844

[33] Arganda-Carreras I, Fernández-González R, Muñoz-Barrutia A, Ortiz-De-Solorzano C. 2010. 3D reconstruction of histological sections: application to mammary gland tissue. Microsc Res Tech 73:1019–1029. doi:10.1002/jemt.20829 20232465

[34] Hindson CM, Chevillet JR, Briggs HA, Gallichotte EN, Ruf IK, Hindson BJ, Vessella RL, Tewari M. 2013. Absolute quantification by droplet digital PCR versus analog real-time PCR. Nat Methods 10:1003–1005. doi:10.1038/nmeth.2633 23995387 PMC4118677

[35] Chung HK, Hattler JB, Narola J, Babbar H, Cai Y, Abdel-Mohsen M, Kim WK. 2022. Development of droplet digital PCR-based assays to quantify HIV proviral and integrated DNA in brain tissues from viremic individuals with encephalitis and virally suppressed aviremic individuals. Microbiol Spectr 10:e0085321. doi:10.1128/spectrum.00853-21 35019681 PMC8754137

[36] Cochrane CR, Angelovich TA, Byrnes SJ, Waring E, Guanizo AC, Trollope GS, Zhou J, Vue J, Senior L, Wanicek E, Eddine JJ, Gartner MJ, Jenkins TA, Gorry PR, Brew BJ, Lewin SR, Estes JD, Roche M, Churchill MJ. 2022. Intact HIV proviruses persist in the brain despite viral suppression with ART. Ann Neurol 92:532–544. doi:10.1002/ana.26456 35867351 PMC9489665

[37] Zankharia U, Yi Y, Lu F, Vladimirova O, Karisetty BC, Wikramasinghe J, Kossenkov A, Collman RG, Lieberman PM. 2024. HIV-induced RSAD2/viperin supports sustained infection of monocyte-derived macrophages. J Virol 98:1–19. doi:10.1128/jvi.00863-24 PMC11494996 39258908

[38] Eugenin EA, Osiecki K, Lopez L, Goldstein H, Calderon TM, Berman JW. 2006. CCL2/monocyte chemoattractant protein-1 mediates enhanced transmigration of human immunodeficiency virus (HIV)-infected leukocytes across the blood-brain barrier: a potential mechanism of HIV-CNS invasion and NeuroAIDS. J Neurosci 26:1098–1106. doi:10.1523/JNEUROSCI.3863-05.2006 16436595 PMC6674577

[39] Kelder W, McArthur JC, Nance-Sproson T, McClernon D, Griffin DE. 1998. Beta-chemokines MCP-1 and RANTES are selectively increased in cerebrospinal fluid of patients with human immunodeficiency virus-associated dementia. Ann Neurol 44:831–835. doi:10.1002/ana.410440521 9818943

[40] Yamamoto M, Guo DH, Hernandez CM, Stranahan AM. 2019. Endothelial Adora2a activation promotes blood-brain barrier breakdown and cognitive impairment in mice with diet-induced insulin resistance art. J Neurosci 39:4179–4192. doi:10.1523/JNEUROSCI.2506-18.2019 30886019 PMC6529868

[41] Gustin C, Van Steenbrugge M, Raes M. 2008. LPA modulates monocyte migration directly and via LPA-stimulated endothelial cells. Am J Physiol Cell Physiol 295:C905–14. doi:10.1152/ajpcell.00544.2007 18632732

[42] Canonigo-Balancio AJ, Fos C, Prod’homme T, Bécart S, Altman A. 2009. SLAT/Def6 plays a critical role in the development of Th17 cell-mediated experimental autoimmune encephalomyelitis. J Immunol 183:7259–7267. doi:10.4049/jimmunol.0902573 19915062 PMC2821872

[43] Kannan AK, Kim DG, August A, Bynoe MS. 2015. Itk signals promote neuroinflammation by regulating CD4+ T-cell activation and trafficking. J Neurosci 35:221–233. doi:10.1523/JNEUROSCI.1957-14.2015 25568116 PMC4287144

[44] Byrd DA, Fellows RP, Morgello S, Franklin D, Heaton RK, Deutsch R, Atkinson JH, Clifford DB, Collier AC, Marra CM, Gelman B, McCutchan JA, Duarte NA, Simpson DM, McArthur J, Grant I, Group C. 2011. Neurocognitive impact of substance use in HIV infection. J Acquir Immune Defic Syndr 58:154–162. doi:10.1097/QAI.0b013e318229ba41 21725250 PMC3183737

[45] Hauser KF, Fitting S, Dever SM, Podhaizer EM, Knapp PE. 2012. Opiate drug use and the pathophysiology of neuroAIDS. Curr HIV Res 10:435–452. doi:10.2174/157016212802138779 22591368 PMC3431547

[46] Massanella M, Gianella S, Schrier R, Dan JM, Pérez-Santiago J, Oliveira MF, Richman DD, Little SJ, Benson CA, Daar ES, Dube MP, Haubrich RH, Smith DM, Morris SR. 2015. Methamphetamine use in HIV-infected individuals affects T-cell function and viral outcome during suppressive antiretroviral therapy. Sci Rep 5:13179. doi:10.1038/srep13179 26299251 PMC4547398

[47] Rippeth JD, Heaton RK, Carey CL, Marcotte TD, Moore DJ, Gonzalez R, Wolfson T, Grant I, Group TH. 2004. Methamphetamine dependence increases risk of neuropsychological impairment in HIV infected persons. J Inter Neuropsych Soc 10:1–14. doi:10.1017/S1355617704101021 14751002

[48] Peng X, Mosser DM, Adler MW, Rogers TJ, Meissler JJ Jr, Eisenstein TK. 2000. Morphine enhances interleukin-12 and the production of other pro-inflammatory cytokines in mouse peritoneal macrophages. J Leukoc Biol 68:723–728. 11073113

[49] Zeitz KP, Malmberg AB, Gilbert H, Basbaum AI. 2001. Reduced development of tolerance to the analgesic effects of morphine and clonidine in PKCγ mutant mice. PAIN 94:245–253. doi:10.1016/S0304-3959(01)00353-0 11731061

[50] Cheng WS, Garfein RS, Semple SJ, Strathdee SA, Zians JK, Patterson TL. 2010. Binge use and sex and drug use behaviors among HIV(-), heterosexual methamphetamine users in San Diego. Subst Use Misuse 45:116–133. doi:10.3109/10826080902869620 20025442 PMC2861916

[51] Kesby JP, Fields JA, Chang A, Coban H, Achim CL, Semenova S, Group T. 2018. Effects of HIV-1 TAT protein and methamphetamine exposure on visual discrimination and executive function in mice. Behav Brain Res 349:73–79. doi:10.1016/j.bbr.2018.04.046 29709610 PMC6320247

[52] Saylor D, Dickens AM, Sacktor N, Haughey N, Slusher B, Pletnikov M, Mankowski JL, Brown A, Volsky DJ, McArthur JC. 2016. HIV-associated neurocognitive disorder — pathogenesis and prospects for treatment. Nat Rev Neurol 12:234–248. doi:10.1038/nrneurol.2016.27 26965674 PMC4937456

[53] Heaton RK, Clifford DB, Franklin DR, Woods SP, Ake C, Vaida F, Ellis RJ, Letendre SL, Marcotte TD, Atkinson JH, et al.. 2010. HIV-associated neurocognitive disorders persist in the era of potent antiretroviral therapy. Neurology (ECronicon) 75:2087–2096. doi:10.1212/WNL.0b013e318200d727 PMC2995535 21135382

[54] Clifford DB. 2017. HIV-associated neurocognitive disorder. Curr Opin Infect Dis 30:117–122. doi:10.1097/QCO.0000000000000328 27798498 PMC5382956

[55] Conant K, Garzino-Demo A, Nath A, McArthur JC, Halliday W, Power C, Gallo RC, Major EO. 1998. Induction of monocyte chemoattractant protein-1 in HIV-1 Tat-stimulated astrocytes and elevation in AIDS dementia. Proc Natl Acad Sci USA 95:3117–3121. doi:10.1073/pnas.95.6.3117 9501225 PMC19704

[56] Hong S, Banks WA. 2015. Role of the immune system in HIV-associated neuroinflammation and neurocognitive implications. Brain Behav Immun 45:1–12. doi:10.1016/j.bbi.2014.10.008 25449672 PMC4342286

[57] Thompson KA, Cherry CL, Bell JE, McLean CA. 2011. Brain cell reservoirs of latent virus in presymptomatic HIV-infected individuals. Am J Pathol 179:1623–1629. doi:10.1016/j.ajpath.2011.06.039 21871429 PMC3181362

[58] Wang H, Sun J, Goldstein H. 2008. Human immunodeficiency virus type 1 infection increases the in vivo capacity of peripheral monocytes to cross the blood-brain barrier into the brain and the in vivo sensitivity of the blood-brain barrier to disruption by lipopolysaccharide. J Virol 82:7591–7600. doi:10.1128/JVI.00768-08 18508884 PMC2493310

[59] Abbott NJ, Patabendige AAK, Dolman DEM, Yusof SR, Begley DJ. 2010. Structure and function of the blood–brain barrier. Neurobiol Dis 37:13–25. doi:10.1016/j.nbd.2009.07.030 19664713

[60] Wu D, Chen Q, Chen X, Han F, Chen Z, Wang Y. 2023. The blood–brain barrier: structure, regulation and drug delivery. Sig Transduct Target Ther 8:217. doi:10.1038/s41392-023-01481-w PMC10212980 37231000

[61] Banks WA, Erickson MA. 2010. The blood-brain barrier and immune function and dysfunction. Neurobiol Dis 37:26–32. doi:10.1016/j.nbd.2009.07.031 19664708

[62] Dutta R, Roy S. 2015. Chronic morphine and HIV-1 Tat promote differential central nervous system trafficking of CD3+ and Ly6C+ immune cells in a murine Streptococcus pneumoniae infection model. J Neuroinflammation 12:120. doi:10.1186/s12974-015-0341-5 26087960 PMC4490693

[63] Marchetti G, Tincati C, Silvestri G. 2013. Microbial translocation in the pathogenesis of HIV infection and AIDS. Clin Microbiol Rev 26:2–18. doi:10.1128/CMR.00050-12 23297256 PMC3553668

[64] Rempel H, Sun B, Calosing C, Pillai SK, Pulliam L. 2010. Interferon-α drives monocyte gene expression in chronic unsuppressed HIV-1 infection. AIDS 24:1415–1423. doi:10.1097/QAD.0b013e32833ac623 20495440 PMC2991092

[65] van Pul L, van Dort KA, Girigorie AF, Maurer I, Harskamp AM, Kootstra NA. 2024. Human immunodeficiency virus-induced interferon-stimulated gene expression is associated with monocyte activation and predicts viral load. Open Forum Infect Dis 11:ofae434. doi:10.1093/ofid/ofae434 39104769 PMC11298257

[66] Klatt NR, Funderburg NT, Brenchley JM. 2013. Microbial translocation, immune activation, and HIV disease. Trends Microbiol 21:6–13. doi:10.1016/j.tim.2012.09.001 23062765 PMC3534808

[67] Brenchley JM, Price DA, Schacker TW, Asher TE, Silvestri G, Rao S, Kazzaz Z, Bornstein E, Lambotte O, Altmann D, Blazar BR, Rodriguez B, Teixeira-Johnson L, Landay A, Martin JN, Hecht FM, Picker LJ, Lederman MM, Deeks SG, Douek DC. 2006. Microbial translocation is a cause of systemic immune activation in chronic HIV infection. Nat Med 12:1365–1371. doi:10.1038/nm1511 17115046

[68] Brenchley JM, Douek DC. 2012. Microbial translocation across the GI tract. Annu Rev Immunol 30:149–173. doi:10.1146/annurev-immunol-020711-075001 22224779 PMC3513328

[69] Dohgu S, Fleegal-DeMotta MA, Banks WA. 2011. Lipopolysaccharide-enhanced transcellular transport of HIV-1 across the blood-brain barrier is mediated by luminal microvessel IL-6 and GM-CSF. J Neuroinflammation 8:167. doi:10.1186/1742-2094-8-167 22129063 PMC3260201

[70] Kanmogne GD, Schall K, Leibhart J, Knipe B, Gendelman HE, Persidsky Y. 2007. HIV-1 gp120 compromises blood-brain barrier integrity and enhances monocyte migration across blood-brain barrier: implication for viral neuropathogenesis. J Cereb Blood Flow Metab 27:123–134. doi:10.1038/sj.jcbfm.9600330 16685256 PMC2232899

[71] Dhillon NK, Williams R, Callen S, Zien C, Narayan O, Buch S. 2008. Roles of MCP-1 in development of HIV-dementia. Front Biosci 13:3913–3918. doi:10.2741/2979 18508485 PMC2715276

[72] D’Mello C, Le T, Swain MG. 2009. Cerebral microglia recruit monocytes into the brain in response to tumor necrosis factoralpha signaling during peripheral organ inflammation. J Neurosci 29:2089–2102. doi:10.1523/JNEUROSCI.3567-08.2009 19228962 PMC6666330

[73] Yu Q, Chow EMC, Wong H, Gu J, Mandelboim O, Gray-Owen SD, Ostrowski MA. 2006. CEACAM1 (CD66a) promotes human monocyte survival via a phosphatidylinositol 3-kinase- and AKT-dependent pathway. J Biol Chem 281:39179–39193. doi:10.1074/jbc.M608864200 17071610

[74] Strazza M, Pirrone V, Wigdahl B, Dampier W, Lin W, Feng R, Maubert ME, Weksler B, Romero IA, Couraud PO, Nonnemacher MR. 2016. Prolonged morphine exposure induces increased firm adhesion in an in vitro model of the blood-brain barrier. Int J Mol Sci 17:916. doi:10.3390/ijms17060916 27294916 PMC4926449

[75] Mahajan SD, Aalinkeel R, Sykes DE, Reynolds JL, Bindukumar B, Fernandez SF, Chawda R, Shanahan TC, Schwartz SA. 2008. Tight junction regulation by morphine and HIV-1 tat modulates blood-brain barrier permeability. J Clin Immunol 28:528–541. doi:10.1007/s10875-008-9208-1 18574677

[76] Sharma HS, Sjöquist P-O, Ali SF. 2010. Alterations in blood-brain barrier function and brain pathology by morphine in the rat. Neuroprotective effects of antioxidant H-290/51. Acta Neurochir Suppl 106:61–66. doi:10.1007/978-3-211-98811-4_10 19812922

[77] Sharma HS, Ali SF. 2006. Alterations in blood-brain barrier function by morphine and methamphetamine. Ann N Y Acad Sci 1074:198–224. doi:10.1196/annals.1369.020 17105918

[78] Bowyer JF, Ali S. 2006. High doses of methamphetamine that cause disruption of the blood-brain barrier in limbic regions produce extensive neuronal degeneration in mouse hippocampus. Synapse 60:521–532. doi:10.1002/syn.20324 16952162

[79] Koyanagi Y, Miles S, Mitsuyasu RT, Merrill JE, Vinters HV, Chen IS. 1987. Dual infection of the central nervous system by AIDS viruses with distinct cellular tropisms. Science 236:819–822. doi:10.1126/science.3646751 3646751

[80] Baba M, Miyake H, Okamoto M, Iizawa Y, Okonogi K. 2000. Establishment of a CCR5-expressing T-lymphoblastoid cell line highly susceptible to R5 HIV type 1. AIDS Res Hum Retroviruses 16:935–941. doi:10.1089/08892220050058344 10890354

[81] Flerin NC, Bardhi A, Zheng JH, Korom M, Folkvord J, Kovacs C, Benko E, Truong R, Mota T, Connick E, Jones RB, Lynch RM, Goldstein H. 2019. Establishment of a novel humanized mouse model to investigate in vivo activation and depletion of patient-derived HIV latent reservoirs. J Virol 93:e02051-18. doi:10.1128/JVI.02051-18 30626677 PMC6401459

[82] Pasternak AO, Adema KW, Bakker M, Jurriaans S, Berkhout B, Cornelissen M, Lukashov VV. 2008. Highly sensitive methods based on seminested real-time reverse transcription-PCR for quantitation of human immunodeficiency virus type 1 unspliced and multiply spliced RNA and proviral DNA. J Clin Microbiol 46:2206–2211. doi:10.1128/JCM.00055-08 18463204 PMC2446885

[83] Andrews S. 2010. FastQC: a quality control tool for high throughput sequence data. Available from: http://wwwbioinformaticsbabrahamacuk/projects/fastqc

[84] Martin M. 2011. Cutadapt removes adapter sequences from high-throughput sequencing reads. EMBnet J 17:10. doi:10.14806/ej.17.1.200

[85] Dobin A, Davis CA, Schlesinger F, Drenkow J, Zaleski C, Jha S, Batut P, Chaisson M, Gingeras TR. 2013. STAR: ultrafast universal RNA-seq aligner. Bioinformatics 29:15–21. doi:10.1093/bioinformatics/bts635 23104886 PMC3530905

[86] García-Alcalde F, Okonechnikov K, Carbonell J, Cruz LM, Götz S, Tarazona S, Dopazo J, Meyer TF, Conesa A. 2012. Qualimap: evaluating next-generation sequencing alignment data. Bioinformatics 28:2678–2679. doi:10.1093/bioinformatics/bts503 22914218

[87] Liao Y, Smyth GK, Shi W. 2014. featureCounts: an efficient general purpose program for assigning sequence reads to genomic features. Bioinformatics 30:923–930. doi:10.1093/bioinformatics/btt656 24227677

[88] Robinson MD, McCarthy DJ, Smyth GK. 2010. edgeR: a bioconductor package for differential expression analysis of digital gene expression data. Bioinformatics 26:139–140. doi:10.1093/bioinformatics/btp616 19910308 PMC2796818

