# Supplementary material for: HIV transgenic mouse monocytes display increased in vivo migration across the blood-brain barrier associated with increased expression of genes associated with mononuclear leukocyte movement
Source: J Virol. 2026 Apr 20;100(5):e02063-25. doi: 10.1128/jvi.02063-25 (PMC13185604; doi:10.1128/jvi.02063-25)
Supplement: Supplemental material — Tables S1 to S3; Fig. S1. [file jvi.02063-25-s0001.docx]

**Supplementary Table 1 - DEGs Associated with IPA Migration and Adhesion of Monocytes Pathway Differentially Expressed by Monocytes from PBS-treated JRCCC mice Compared to Monocytes from PBS-treated CCC mice**

| **ID** | **Gene ID** | **Gene Name** | **Expression FC** | **P. Value** | **Function** |
| --- | --- | --- | --- | --- | --- |
| **ENSMUSG00000021250** | FOS | Fos Proto-Oncogene, AP-1 Transcription Factor Subunit | 7.26 | 6.67E-05 | Regulating cell proliferation, transformation and differentiation, AP1 transcription factor |
| **ENSMUSG00000024190** | DUSP1 | Dual Specificity Phosphatase 1 | 6.26 | 6.09E-03 | Negative regulation of monocyte chemotaxis, negative regulation of MAPK cascade |
| **ENSMUSG00000000982** | CCL3 | C-C Motif Chemokine Ligand 3 Like 1 | 3.23 | 3.99E-06 | Increases transmigration of Th1 lymphocytes, leukocyte chemotaxis, monocyte chemotaxis, positive regulation of cell migration |
| **ENSMUSG00000035692** | ISG15 | ISG15 Ubiquitin like Modifier | 3.22 | 1.02E-03 | Post-translational modifier of actin which can be upregulated prior to BBB disruption after traumatic brain injury |
| **ENSMUSG00000025498** | IRF7 | Interferon Regulatory Factor 7 | 3.12 | 1.90E-04 | Mouse IRF7 knockout decreases infiltration of CD4 cells to lungs, defense response to virus, regulation of adaptive immune response |
| **ENSMUSG00000002602** | AXL | AXL Receptor Tyrosine Kinase | 2.80 | 1.07E-04 | Cell migration, vascular endothelial growth factor receptor signaling pathway |
| **ENSMUSG00000035385** | CCL2 | C-C Motif Chemokine Ligand 2 | 2.67 | 2.15E-03 | CCL2 knock out decreases infiltration to mouse brain by monocyte that increased by inflammation of liver, chemotaxis, vascular endothelial growth factor receptor signaling pathway, positive regulation of monocyte chemotaxis |
| **ENSMUSG00000035000** | DPP4 | Dipeptidyl Peptidase 4 | 2.36 | 4.14E-05 | Cell adhesion, endothelial cell migration, regulation of cell-cell adhesion mediated by integrin, increases migration of cultured peripheral blood T cells |
| **ENSMUSG00000052684** | JUN | Jun Proto-Oncogene, AP-1 Transcription Factor Subunit | 2.19 | 5.23E-03 | Cell proliferation, monocyte differentiation, positive regulation of endothelial cell migration, AP1 transcription factor |
| **ENSMUSG00000020178** | ADORA2A | Adenosine A2a Receptor | 2.08 | 7.63E-03 | Negative regulation of vascular permeability, plays a role in pathophysiological conditions such as inflammatory diseases and neurodegenerative disorders |
| **ENSMUSG00000000440** | PPARG | Peroxisome Proliferator Activated Receptor Gamma | 1.98 | 3.88E-03 | Monocyte differentiation, inflammatory response, negative regulation of blood vessel endothelial cell migration |
| **ENSMUSG00000016206** | HLA-G | Major Histocompatibility Complex, Class I, G | 1.9 | 3.78E-04 | Nonclassical HLA class Ib molecule with immunomodulatory and anti-inflammatory properties which enables migration of Treg cells across the BBB |
| **ENSMUSG00000029648** | FLT1 | Fms Related Receptor Tyrosine Kinase 1 | 1.75 | 6.12E-03 | Cell migration, monocyte chemotaxis, positive regulation of cell migration, vascular endothelial growth factor receptor signaling pathway |
| **ENSMUSG00000022048** | DPYSL2 | Dihydropyrimidinase Like 2 | 1.68 | 1.58E-03 | Migration of primary human T lymphocytes |
| **ENSMUSG00000053819** | CAMK2D | Calcium/Calmodulin Dependent Protein Kinase II Delta | 1.67 | 4.43E-03 | Positive regulation of ERK1/2 cascade, CAMK2D decreases transendothelial migration of human peripheral blood monocytes |
| **ENSMUSG00000021262** | EVL | Enah/Vasp-like | 1.67 | 3.87E-03 | Regulates activated T cell trafficking by promoting diapedesis during transendothelial migration |
| **ENSMUSG00000025492** | IFITM3 | Interferon Induced Transmembrane Protein 3 | 1.66 | 1.21E-05 | Defense response to virus |
| **ENSMUSG00000053318** | SLAMF8 | SLAM Family Member 8 | 1.65 | 6.17E-04 | A type I cell surface glycoprotein expressed after activation of macrophages by interferon-gamma or bacteria. |
| **ENSMUSG00000017652** | CD40 | CD40 Molecule | 1.62 | 4.43E-03 | Increase the rate of transmigration of Th1 lymphocytes to central nervous system, regulation of blood vessel endothelial cell migration |
| **ENSMUSG00000026104** | STAT1 | Signal Transducer and Activator of Transcription 1 | 1.60 | 2.62E-03 | Endothelial cell migration, JAK-STAT cascade |
| **ENSMUSG00000023034** | NR4A1 | Nuclear Receptor Subfamily 4 Group A Member 1 | 1.55 | 7.35E-03 | Cellular response to vascular endothelial growth factor, endothelial cell chemotaxis |
| **ENSMUSG00000021451** | SEMA4D | Semaphorin 4D | 1.55 | 8.82E-04 | Cell adhesion, negative regulation of cell adhesion, positive regulation of cell migration |
| **ENSMUSG00000026073** | IL1R2 | Interleukin 1 Receptor Type 2 | -1.91 | 2.58E-03 | Cytokine mediated signaling pathway, negative regulation of IL1a production |
| **ENSMUSG00000041324** | INHBA | Inhibin Subunit Beta A | -2.15 | 9.42E-04 | Activation of inhibin signaling pathway |

**Supplementary Table 2 - DEGs Associated with the IPA Cell Movement of Mononuclear Leukocytes Pathway Differentially Expressed by Monocytes from LPS-treated JRCCC mice Compared to Monocytes from LPS-treated CCC mice**

Genes denoted by * are also significant in the PBS-treated JRCCC vs PBS-treated CCC comparison.

| **ID** | **Gene ID** | **Gene Name** | **Expression FC** | **P. Value** | **Function** |
| --- | --- | --- | --- | --- | --- |
| **ENSMUSG00000000982** | CCL3* | C-C Motif Chemokine Ligand 3 Like 1 | -3.32 | 3.48E-06 | Increases transmigration of Th1 lymphocytes, leukocyte chemotaxis, monocyte chemotaxis, positive regulation of cell migration |
| **ENSMUSG00000052684** | JUN* | Jun Proto-Oncogene, AP-1 Transcription Factor Subunit | -3.43 | 1.35E-05 | Cell proliferation, monocyte differentiation, positive regulation of endothelial cell migration, AP1 transcription factor |
| **ENSMUSG00000053819** | CAMK2D* | Calcium/Calmodulin Dependent Protein Kinase II Delta | -2.64 | 3.38E-05 | Positive regulation of ERK1/2 cascade, CAMK2D decreases transendothelial migration of human peripheral blood monocytes |
| **ENSMUSG00000021250** | FOS* | Fos Proto-Oncogene, AP-1 Transcription Factor Subunit | -6.42 | 1.19E-04 | Regulating cell proliferation, transformation and differentiation, AP1 transcription factor |
| **ENSMUSG00000022048** | DPYSL2* | Dihydropyrimidinase Like 2 | -2.66 | 2.05E-04 | Migration of primary human T lymphocytes |
| **ENSMUSG00000041324** | INHBA* | Inhibin Subunit Beta A | 1.8 | 4.65E-04 | Activation of inhibin signaling pathway |
| **ENSMUSG00000053318** | SLAMF8* | SLAM Family Member 8 | -2.19 | 6.47E-04 | A type I cell surface glycoprotein expressed after activation of macrophages by interferon-gamma or bacteria. |
| **ENSMUSG00000002602** | AXL* | AXL Receptor Tyrosine Kinase | -2.71 | 1.57E-03 | Cell migration, vascular endothelial growth factor receptor signaling pathway |
| **ENSMUSG00000035000** | DPP4* | Dipeptidyl Peptidase 4 | -2.76 | 2.14E-03 | Cell adhesion, endothelial cell migration, regulation of cell-cell adhesion mediated by integrin, increases migration of cultured peripheral blood T cells |
| **ENSMUSG00000035385** | CCL2* | C-C Motif Chemokine Ligand 2 | -1.85 | 2.68E-03 | CCL2 knock out decreases infiltration to mouse brain by monocyte that increased by inflammation of liver, chemotaxis, vascular endothelial growth factor receptor signaling pathway, positive regulation of monocyte chemotaxis |
| **ENSMUSG00000016206** | HLA-G* | Major Histocompatibility Complex, Class I, G | -1.67 | 4.33E-03 | Nonclassical HLA class Ib molecule with immunomodulatory and anti-inflammatory properties which enables migration of Treg cells across the BBB |
| **ENSMUSG00000024190** | DUSP1* | Dual Specificity Phosphatase 1 | -3.92 | 7.42E-03 | Negative regulation of monocyte chemotaxis, negative regulation of MAPK cascade |
| **ENSMUSG00000021262** | EVL | Enah/Vasp-like | -1.68 | 1.16E-02 | Regulates activated T cell trafficking by promoting diapedesis during transendothelial migration |
| **ENSMUSG00000017652** | CD40 | CD40 Molecule | -1.99 | 1.92E-02 | Increase the rate of transmigration of Th1 lymphocytes to central nervous system, regulation of blood vessel endothelial cell migration |
| **ENSMUSG00000031015** | KLRK1 | Killer Cell Lectin-Like Receptor Subfamily K, Member 1 | -2.02 | 4.42E-02 | Encodes NKG2D, plays a role in immune surveillance and helps to detect and eliminate virus -infected cells and tumor cells |
| **ENSMUSG00000023034** | NR4A1 | Nuclear Receptor Subfamily 4 Group A Member 1 | -1.39 | 6.99E-02 | Cell migration involving sprouting angiogenesis, cellular response to vascular endothelial growth factor stimulus, endothelial cell chemotaxis |
| **ENSMUSG00000026073** | IL1R2 | Interleukin 1 Receptor Type 2 | 1.21 | 0.15 | Cytokine mediated signaling pathway, negative regulation of IL1a production |
| **ENSMUSG00000026104** | STAT1 | Signal Transducer and Activator of Transcription 1 | -1.2 | 0.18 | Endothelial cell migration, JAK-STAT cascade |
| **ENSMUSG00000035692** | ISG15 | ISG15 Ubiquitin like Modifier | -1.3 | 0.19 | Post-translational modifier of actin which can be upregulated prior to BBB disruption after traumatic brain injury |
| **ENSMUSG00000029648** | FLT1 | Fms Related Receptor Tyrosine Kinase 1 | 1.37 | 0.2 | Cell migration, monocyte chemotaxis, positive regulation of cell migration, vascular endothelial growth factor receptor signaling pathway |
| **ENSMUSG00000020178** | ADORA2A | Adenosine A2a Receptor | -1.52 | 0.22 | Negative regulation of vascular permeability, plays a role in pathophysiological conditions such as inflammatory diseases and neurodegenerative disorders |
| **ENSMUSG00000052397** | IFITM3 | Interferon-Induced Transmembrane Protein 3 | -1.2 | 0.24 | Prevents viruses from entering the cytoplasm after endocytosis via alterations in the endosomal membrane |
| **ENSMUSG00000024397** | TG | Thyroglobulin | -1.16 | 0.27 | Precursor for T3/T4 production |
| **ENSMUSG00000000440** | PPARG | Peroxisome Proliferator Activated Receptor Gamma | -1.24 | 0.32 | Monocyte differentiation, inflammatory response, negative regulation of blood vessel endothelial cell migration |
| **ENSMUSG00000025498** | IRF7 | Interferon Regulatory Factor 7 | -1.21 | 0.36 | Mouse IRF7 knockout decreases infiltration of CD4 cells to lungs, defense response to virus, regulation of adaptive immune response |
| **ENSMUSG00000021451** | SEMA4D | Semaphorin 4D | -1.08 | 0.46 | Cell adhesion, negative regulation of cell adhesion, positive regulation of cell migration |

**Supplementary Table 3 - DEGs Associated with the IPA Cell Movement of Mononuclear Leukocytes Pathway Differentially Expressed by Monocytes from LPS-treated JRCCC mice Compared to Monocytes from PBS-treated JRCCC mice**

| **ID** | **Gene ID** | **Gene Name** | **Expression FC** | **P. Value** | **Function** |
| --- | --- | --- | --- | --- | --- |
| **ENSMUSG00000022791** | TNK2 | Tyrosine Kinase Non-Receptor 2 | 2.94 | 8.21E-09 | Decreases motility of primary culture CD4 cells that involved ICAM2 protein |
| **ENSMUSG00000026581** | SELL | Selectin L | -2.07 | 2.21E-08 | Downregulation increases trafficking of T lymphocytes to inflamed tissue, cell adhesion, leukocyte migration |
| **ENSMUSG00000024349** | TMEM173 (STING1) | Stimulator of Interferon Response cGAMP Interactor 1 | -2.91 | 4.99E-08 | Knockout decreases transendothelial migration of co-cultured mouse Th1 cells |
| **ENSMUSG00000004446** | BID | BH3 Interacting Domain Death Agonist | -2.03 | 1.42E-07 | Pro-apoptotic member of the Bcl-2 protein family which regulates cell death and can counter the protective anti-apoptotic effect of BCL-2 |
| **ENSMUSG00000022048** | DPYSL2 | Dihydropyrimidinase Like 2 | -6.30 | 1.63E-07 | Migration of primary human T lymphocytes |
| **ENSMUSG00000028970** | ABCB1B | ATP-Binding Cassette, Sub-Family B member 1B | -3.27 | 3.40E-07 | ABCB1B + ABCC1 are necessary for migration of T lymphocytes, establishment of endothelial blood-brain barrier, response to LPS |
| **ENSMUSG00000022892** | APP | Amyloid Beta Precursor Protein | 1.63 | 4.57E-07 | Increase transmigration of co-cultured peripheral blood monocytes, cell adhesion, positive regulation of monocyte chemotaxis |
| **ENSMUSG00000020717** | PECAM1 | platelet and endothelial cell adhesion molecule 1 | -2.81 | 5.48E-07 | Increases adhesion of T lymphocytes that is mediated by ITGB1 protein, necessary for transendothelial migration of co-culture monocytes from human peripheral blood, endothelial cell migration, maintenance of blood-brain barrier, positive regulation of cell migration |
| **ENSMUSG00000001029** | ICAM2 | Intercellular Adhesion Molecule 2 | -3.89 | 5.98E-07 | Decreases adhesion of nasal polyp endothelial cells and T lymphocytes from human peripheral blood, cell adhesion |
| **ENSMUSG00000002602** | AXL | AXL Receptor Tyrosine Kinase | -7.30 | 1.57E-06 | Cell migration, vascular endothelial growth factor receptor signaling pathway |
| **ENSMUSG00000021948** | PRKCD | Protein Kinase C, Delta | -1.80 | 1.81E-06 | Knockout increase infiltration to mouse lungs tissue by plasma cells, cell chemotaxis |
| **ENSMUSG00000054065** | PKP3 | Plakophilin 3 | -4.10 | 1.82E-06 | Cellular adhesion |
| **ENSMUSG00000055447** | CD47 | CD47 molecule | 1.70 | 2.02E-06 | Rat CD47 increases transmigration of monocytes to brain endothelial cells, is necessary for transendothelial migration of monocytes, cell migration, positive regulation of monocyte extravasation, positive regulation of cell-cell adhesion, monocyte aggregation |
| **ENSMUSG00000036006** | RIPOR2 (Fam65b) | RHO Family Interacting Cell Polarization Regulator 2 | 2.55 | 2.26E-06 | Decrease migration by T cells, negative regulation of cell adhesion |
| **ENSMUSG00000031015** | SWAP70 | SWA-70 Protein | -2.04 | 2.72E-06 | Knockout decreases migration of B cells to mouse lymph nodes, negative regulation of cell-cell adhesion |
| **ENSMUSG00000019843** | FYN | FYN Proto-Oncogene, Src Family Tyrosine Kinase | -3.41 | 2.96E-06 | Inhibition of FYN increases chemotaxis from human T lymphocytes in cultured that is increased by CCR7 protein, vascular endothelial growth factor receptor signaling pathway |
| **ENSMUSG00000025701** | ALOX5 | Arachidonate 5-Lipoxygenase | 3.16 | 3.06E-06 | Mouse Alox5 is necessary for migration of T lymphocytes, leukocyte chemotaxis involved in inflammatory response, leukocyte migration involved in inflammatory response, negative regulation of vascular would healing |
| **ENSMUSG00000030403** | VASP | Vasodilator-Stimulated Phosphoprotein | 2.32 | 4.50E-06 | Knockout in activated T cells from mouse decreases trafficking of activated T cells to mouse lymph nodes, spleen and CNS |
| **ENSMUSG00000021262** | EVL | Enah/Vasp-like | -3.53 | 5.18E-06 | Regulates activated T cell trafficking by promoting diapedesis during transendothelial migration |
| **ENSMUSG00000002257** | CEF6 | DEF6 Guanine Nucleotide Exchange Factor | 1.57 | 5.87E-06 | Knockout decreases infiltration to CNS |
| **ENSMUSG00000028163** | NKFB1 | Nuclear Factor Kappa B Subunit 1 | -1.53 | 7.04E-06 | Response to LPS |
| **ENSMUSG00000052397** | EZR | Ezrin | 1.84 | 7.18E-06 | Increases rate of chemotactic migration of T lymphocytes in culture, establishment of endothelial barrier, leukocyte cell-cell adhesion |
| **ENSMUSG00000024397** | AIF1 | Allograft Inflammatory Factor 1 | -2.11 | 7.55E-06 | Increases migration by T cells, positive regulation of cell migration, positive regulation of monocyte chemotaxis, positive regulation of mononuclear cell migration |
| **ENSMUSG00000020395** | ITK | IL2 Inducible T Cell Kinase | 2.85 | 7.82E-06 | Knockout decreases migration of CD4 T cells to CNS |
| **ENSMUSG00000037337** | MAP4K1 | Mitogen-Activated Protein Kinase Kinase Kinase Kinase 1 | -1.74 | 8.48E-06 | Knockout increases adhesion of mouse ICAM1 and mouse T lymphocytes in culture |
| **ENSMUSG00000049299** | TRAPPC1 | Trafficking Protein Particle Complex Subunit 1 | -1.62 | 8.71E-06 | Knockout in thymic epithelial cells increase infiltration to kidney by lymphocytes |
| **ENSMUSG00000023034** | NR4A1 | Nuclear Receptor Subfamily 4 Group A Member 1 | -3.04 | 8.98E-06 | Cell migration involving sprouting angiogenesis, cellular response to vascular endothelial growth factor stimulus, endothelial cell chemotaxis |
| **ENSMUSG00000038260** | TRPM4 | Transient Receptor Potential Cation Channel Subfamily M Member 4 | -5.94 | 9.20E-06 | Increases motility of Th1 cells and decrease motility of Th2 cells, positive regulation of vasoconstriction |
| **ENSMUSG00000046223** | PLAUR | Plasminogen Activator, Urokinase Receptor | 2.18 | 1.16E-05 | Decreases migration of monocytes, regulation of cell adhesion |
| **ENSMUSG00000074886** | GRK6 | G Protein-Coupled Receptor Kinase 6 | 1.85 | 1.17E-05 | Knockout decreases migration of mouse T lymphocytes in cell culture, cellular response to LPS |
| **ENSMUSG00000018476** | KDM6B | KDM1 Lysine (K)-Specific Demethylase 6B | 2.40 | 1.24E-05 | Knockout decreases infiltration to mouse lungs by monocytes |
| **ENSMUSG00000037860** | AIM2 | Absent in Melanoma 2 | -2.12 | 1.26E-05 | A cytosolic dsDNA sensor that has been broadly studied for its role in inflammasome assembly |
| **ENSMUSG00000037902** | SIRPA | Signal-Regulatory Protein Alpha | -1.58 | 1.45E-05 | Decreases transmigration of monocytes to brain endothelial cells, cell adhesion, response to LPS, negative regulation of ERK1/2 cascade |
| **ENSMUSG00000002603** | TGFB1 | Transforming Growth Factor Beta 1 | -1.59 | 1.45E-05 | Decreases transmigration of cultured human monocytes from matrigel, negative regulation of blood vessel endothelial cell migration, negative regulation of cell-cell adhesion, positive regulation of cell migration, positive regulation of chemotaxis |
| **ENSMUSG00000029213** | COMMD8 | COMM Domain Containing 8 | -1.51 | 1.66E-05 | Decrease migration of follicular B cells from spleen in cell culture |
| **ENSMUSG00000031934** | PANX1 | Pannexin 1 | -2.05 | 1.68E-05 | Panx1-mediated ATP release is involved in inflammasome activation and neutrophil/macrophage chemotaxis and inducing and propagating inflammation |
| **ENSMUSG00000027312** | ATRN | Attractin | 1.88 | 1.82E-05 | Mediates spreading of monocytes |
| **ENSMUSG00000038037** | SOCS1 | Suppressor of Cytokine Signaling 1 | 3.35 | 1.84E-05 | Knockout increases infiltration in mouse pancreas by T lymphocytes, cellular response to vascular endothelial growth factor stimulus, response to LPS |
| **ENSMUSG00000027808** | SERP1 | Stress Associated Endoplasmic Reticulum Protein 1 | -1.75 | 1.98E-05 | Decreases invasion of T cell |
| **ENSMUSG00000030265** | KRAS | KRAS Proto-Oncogene, GTPase | 1.64 | 2.02E-05 | GTPase part of RAS/MAPK pathway which regulates cell growth, maturation, and death |
| **ENSMUSG00000030067** | FOXP1 | Forkhead Box P1 | -1.85 | 2.19E-05 | Knockout increase migration of CD4T cells from mouse spleen to B cell follicle, endothelial cell activation, positive regulation of endothelial cell migration |
| **ENSMUSG00000022901** | CD86 | CD86 Molecule | -2.82 | 2.79E-05 | CD40 signaling pathway, cellular response ot LPS, decreases transendothelial migration in cell culture |
| **ENSMUSG00000039936** | PIK3CD | Phosphatidylinositol-4,5-Bisphosphate 3-Kinase Catalytic Subunit Delta | -1.59 | 3.13E-05 | Cell migration, chemotaxis, positive regulation of cell migration, positive regulation of vascular endothelial growth factor signaling pathway, decreases migration of NK cells to inflamed mouse peritoneum |
| **ENSMUSG00000016206** | HLA-G | Major Histocompatibility Complex, Class I, G | -2.45 | 3.39E-05 | Nonclassical HLA class Ib molecule with immunomodulatory and anti-inflammatory properties which enables migration of Treg cells across the BBB |
| **ENSMUSG00000023951** | VEGFA | Vascular Endothelial Growth Factor A | 1.88 | 6.20E-05 | Decreases migration of monocytes that is increased by VEGFA protein, cell migration, endothelial cell migration |
| **ENSMUSG00000020178** | ADORA2A | Adenosine A2a Receptor | -5.01 | 8.76E-05 | Negative regulation of vascular permeability, plays a role in pathophysiological conditions such as inflammatory diseases and neurodegenerative disorders |
| **ENSMUSG00000037523** | MAVS | Mitochondrial Antiviral Signaling Protein | -2.60 | 8.88E-05 | Knockout increases infiltration to mouse peribronchial space in lungs |
| **ENSMUSG00000020437** | MYO1G | Myosin 1G | -1.64 | 9.17E-05 | Knockout decreases spread of migration of B lymphocytes, T cell migration |
| **ENSMUSG00000026104** | STAT1 | Signal Transducer and Activator of Transcription 1 | -2.00 | 1.15E-04 | Endothelial cell migration, JAK-STAT cascade |
| **ENSMUSG00000052593** | ADAM17 | ADAM Metallopeptidase Domain 17 | -1.51 | 1.17E-04 | Decreases diapedesis of human peripheral blood monocytes, cell adhesion, cell motility, positive regulation of blood vessel endothelial cell migration, regulation of leukocyte chemotaxis |
| **ENSMUSG00000009647** | MCU | Mitochondrial Calcium Uniporter | 1.62 | 1.46E-04 | Knockout in T cell decreases migration of activated mouse CD4 T cells |
| **ENSMUSG00000013663** | PTEN | Phosphatase and Tensin Homolog | 1.62 | 1.78E-04 | Knockout increases chemotaxis of T lymphocytes in mice, cell migration, cell motility, endothelial cell migration |
| **ENSMUSG00000039217** | Il18 | Interleukin 18 | -4.99 | 2.04E-04 | Human IL18 increases invasiveness of T lymphocytes expressing hCD4 protein from RA patients, positive regulation of vascular permeability, regulation of cell adhesion |
| **ENSMUSG00000038642** | CTSS | Casthepsin S | -2.98 | 2.27E-04 | Increases transmigration of monocytes, knockout decreases transmigration of monocytes in subendothelium |
| **ENSMUSG00000026193** | FN1 | Fibronectin 1 | -7.08 | 2.44E-04 | Increases migration of T cells, cell adhesion, cellular response to LPS, positive regulation of cell migration |
| **ENSMUSG00000074272** | CEACAM1 | CEA Cell Adhesion Molecule 1 | 2.17 | 3.36E-04 | Increases migration of monocytes, cell adhesion, cell migration, regulation of endothelial cell migration |
| **ENSMUSG00000024778** | FAS | FAS Cell Surface Death Receptor | -1.75 | 3.54E-04 | Increases adhesion of purified T lymphocytes |
| **ENSMUSG00000032035** | EST1 | ETS Proto-Oncogene 1, Transcription Factor | 1.93 | 4.06E-04 | Knockout decreases recruitment of T lymphocytes to mouse wall of thoracic aorta, cell motility, positive regulation of blood vessel endothelial cell migration, positive regulation of cell migration, |
| **ENSMUSG00000052821** | CYSLTR1 | Cysteinyl Leukotriene Receptor 1 | -2.69 | 4.20E-04 | Chemotaxis, positive regulation of vasoconstriction, increases chemotaxis of mouse gamma delta T cells |
| **ENSMUSG00000001741** | IL16 | Interleukin 16 | 1.59 | 4.66E-04 | HumanIL16 increases chemotaxis of T lymphocytes, leukocyte chemotaxis, positive regulation of chemokine production |
| **ENSMUSG00000038843** | GCNT1 | Glucosaminyl (N-acetyl) Transferase 1 | -1.69 | 5.76E-04 | Cell adhesion molecule production, knockout decreases rolling of Th1 lymphocytes |
| **ENSMUSG00000059552** | TRP53 | Tumor Protein p53 | -1.50 | 5.79E-04 | Transcription factor that induces cell cycle arrest, DNA repair or apoptosis and acts as a tumor suppressor in many tumor types |
| **ENSMUSG00000015452** | AGER | Advanced Glycosylation End-product Specific Receptor | 3.35 | 7.96E-04 | Increases migration of primary human monocytes in cell culture, microglia cell activation, negative regulation of endothelial cell migration, positive regulation of monocyte extravasation |
| **ENSMUSG00000059714** | FLOT1 | Flotillin 1 | 1.51 | 1.08E-03 | Decrease migration of human peripheral T lymphocytes in culture, regulation of cell-cell adhesion |
| **ENSMUSG00000025225** | NFKB2 | Nuclear Factor Kappa B Subunit 2 | -1.53 | 1.20E-03 | Gene knockout decreases migration of B lymphocytes to lymphoid |
| **ENSMUSG00000037706** | Cd81 | CD81 molecule | 1.60 | 1.28E-03 | Increase migration of human peripheral blood NK cells, positive regulation of CD4 molecules, regulation of cell motility |
| **ENSMUSG00000027360** | HDC | Histidine Decarboxylase | 2.18 | 1.50E-03 | Generates histamine, is highly expressed in CD11b+Gr-1+ myeloid cells critical for modulating infection, inflammation and tumorigenesis |
| **ENSMUSG00000019842** | TRAF3IP2 | TRAF3 Interacting Protein 2 | -1.77 | 2.36E-03 | Cytoplasmic adaptor molecule and activator of the transcription factors NF-κB and AP-1 |
| **ENSMUSG00000026814** | ENG | Endoglin | -2.63 | 2.38E-03 | Knockout decreases transendothelial migration of monocytes |
| **ENSMUSG00000022584** | Ly6a | Lymphocyte Antigen 6 Family Member A | -1.56 | 2.63E-03 | Glycosylphosphatidylinositol (GPI)-anchored protein binds AAV to facilitate high BBB transport |
| **ENSMUSG00000031861** | LPAR2 | Lysophosphatidic Acid Receptor 2 | 1.68 | 2.69E-03 | Inhibition decreases chemoattraction of human peripheral blood monocytes, positive regulation of MAPK cascade |
| **ENSMUSG00000067235** | HLA-A | Major Histocompatibility Complex, Class I, A | 5.66 | 2.87E-03 | Antigen presentation |
| **ENSMUSG00000042770** | HEBP1 | Heme Binding Protein 1 | -1.72 | 3.67E-03 | Increases chemotaxis of monocytes |
| **ENSMUSG00000026471** | MR1 | Major Histocompatibtility Ribonucleoprotein 1 | -2.20 | 4.00E-03 | Knockout decreases recruitment of CD4T cells to lungs |
| **ENSMUSG00000050737** | PTGES | Prostaglandin-Endoperoxide Synthase 2 | 2.10 | 4.30E-03 | Maintain of blood-brain barrier, positive regulation of cell migration, positive regulation of vasoconstriction, positive regulation of vascular endothelial growth factor production |
| **ENSMUSG00000002985** | APOE | Apolipoprotein E | -2.71 | 4.35E-03 | Negative regulation of endothelial cell migration |
| **ENSMUSG00000000440** | PPARG | Peroxisome Proliferator Activated Receptor Gamma | -1.96 | 4.65E-03 | Monocyte differentiation, inflammatory response, negative regulation of blood vessel endothelial cell migration |
| **ENSMUSG00000037966** | NINJ1 | Ninjurin-1 | -1.63 | 5.63E-03 | Neutralization decreases migration of monocytes |
| **ENSMUSG00000024399** | LTB | Lymphotoxin Beta | 2.15 | 6.00E-03 | Increased infiltration to liver by T lymphocytes |
| **ENSMUSG00000035448** | CCR3 | C-C Motif Chemokine Receptor 3 | -3.78 | 6.57E-03 | Cell adhesion, cel chemotaxis, ERK1/2 cascade, positive regulation of endothelial cell proliferation, chemokine-mediated signaling pathway |
| **ENSMUSG00000023274** | CD4 | CD4 Molecule | -2.12 | 7.36E-03 | Positive regulation if monocytes differentiation, cell adhesion, positive regulation of MAPK cascade, necessary for migration of lymphocytes |
| **ENSMUSG00000055148** | KLF2 | KLF Transcription Factor 2 | -1.74 | 8.90E-03 | Migration of T lymphocytes |

**Supplementary Figure 1 – Heatmap of the DEGs Associated with the IPA Cell Movement of Mononuclear Leukocytes Pathway Differentially Expressed by Monocytes from LPS-treated JRCCC mice Compared to Monocytes from LPS-treated CCC mice**


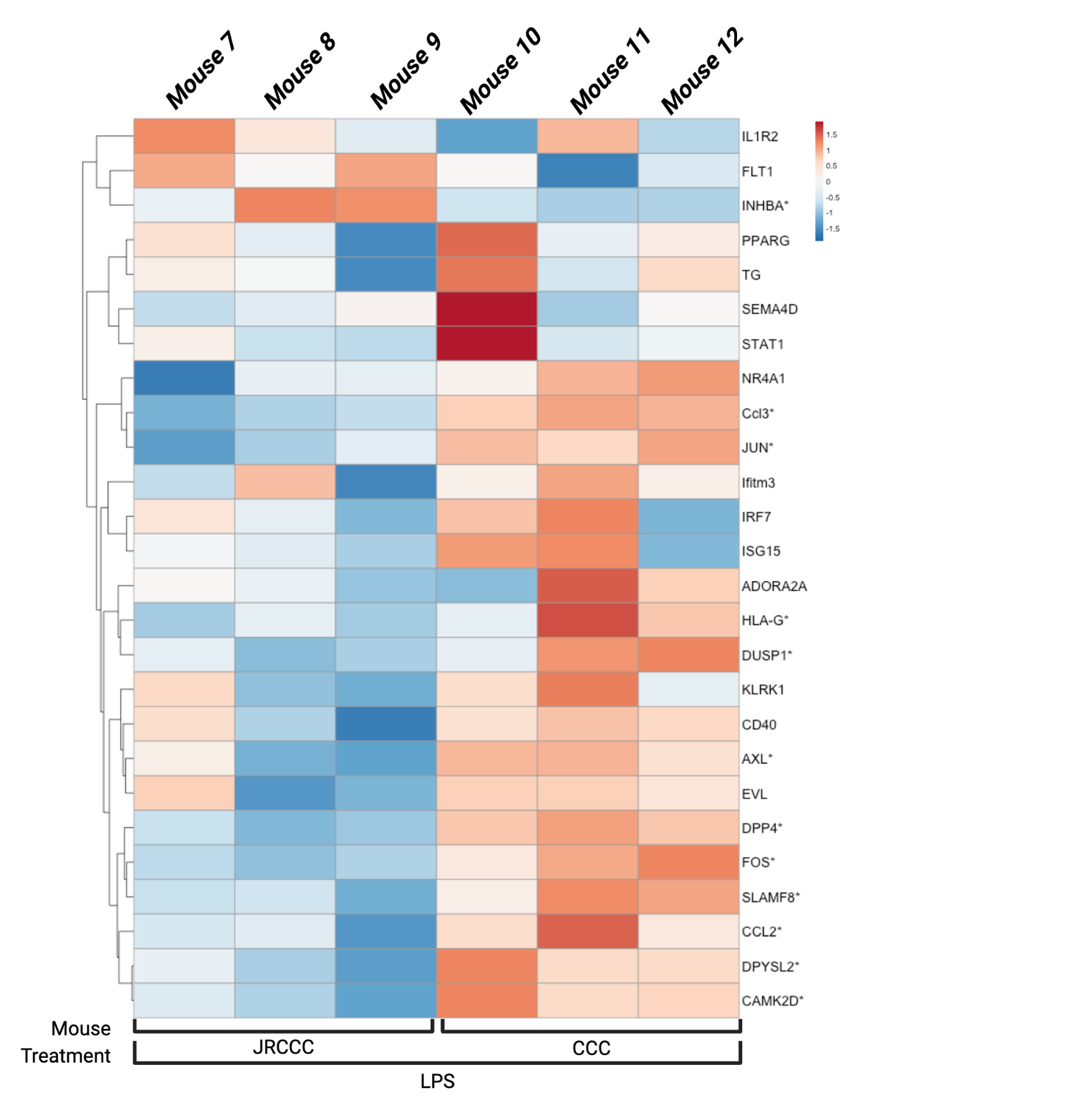
Genes denoted by * are also significant in the PBS-treated JRCCC vs PBS-treated CCC comparison.
